# Acute aerobic exercise reveals that FAHFAs distinguish the metabolomes of overweight and normal-weight runners

**DOI:** 10.1172/jci.insight.158037

**Published:** 2022-04-08

**Authors:** Alisa B. Nelson, Lisa S. Chow, David B. Stagg, Jacob R. Gillingham, Michael D. Evans, Meixia Pan, Curtis C. Hughey, Chad L. Myers, Xianlin Han, Peter A. Crawford, Patrycja Puchalska

**Affiliations:** 1 Division of Molecular Medicine, Department of Medicine,; 2 Bioinformatics and Computational Biology Program,; 3 Division of Diabetes, Endocrinology and Metabolism, Department of Medicine,; 4 Department of Biochemistry, Molecular Biology, and Biophysics, and; 5 Biostatistical Design and Analysis Center, Clinical and Translational Science Institute, University of Minnesota, Minneapolis, Minnesota, USA.; 6 Barshop Institute for Longevity and Aging Studies and; 7 Department of Medicine - Diabetes, University of Texas Health Science Center at San Antonio, San Antonio, Texas, USA.; 8 Department of Integrative Biology and Physiology and; 9 Department of Computer Science and Engineering, University of Minnesota, Minneapolis, Minnesota, USA.

**Keywords:** Metabolism, Adipose tissue, Diabetes, Obesity

## Abstract

**Background:**

Responses of the metabolome to acute aerobic exercise may predict maximum oxygen consumption (VO_2_max) and longer-term outcomes, including the development of diabetes and its complications.

**Methods:**

Serum samples were collected from overweight/obese trained (OWT) and normal-weight trained (NWT) runners prior to and immediately after a supervised 90-minute treadmill run at 60% VO_2_max (NWT = 14, OWT = 11) in a cross-sectional study. We applied a liquid chromatography high-resolution–mass spectrometry–based untargeted metabolomics platform to evaluate the effect of acute aerobic exercise on the serum metabolome.

**Results:**

NWT and OWT metabolic profiles shared increased circulating acylcarnitines and free fatty acids (FFAs) with exercise, while intermediates of adenine metabolism, inosine, and hypoxanthine were strongly correlated with body fat percentage and VO_2_max. Untargeted metabolomics-guided follow-up quantitative lipidomic analysis revealed that baseline levels of fatty acid esters of hydroxy fatty acids (FAHFAs) were generally diminished in the OWT group. FAHFAs negatively correlated with visceral fat mass and HOMA-IR. Strikingly, a 4-fold decrease in FAHFAs was provoked by acute aerobic running in NWT participants, an effect that negatively correlated with circulating IL-6; these effects were not observed in the OWT group. Machine learning models based on a preexercise metabolite profile that included FAHFAs, FFAs, and adenine intermediates predicted VO_2_max.

**Conclusion:**

These findings in overweight human participants and healthy controls indicate that exercise-provoked changes in FAHFAs distinguish normal-weight from overweight participants and could predict VO_2_max. These results support the notion that FAHFAs could modulate the inflammatory response, fuel utilization, and insulin resistance.

**Trial registration:**

ClinicalTrials.gov, NCT02150889.

**Funding:**

NIH DK091538, AG069781, DK098203, TR000114, UL1TR002494.

## Graphical abstract

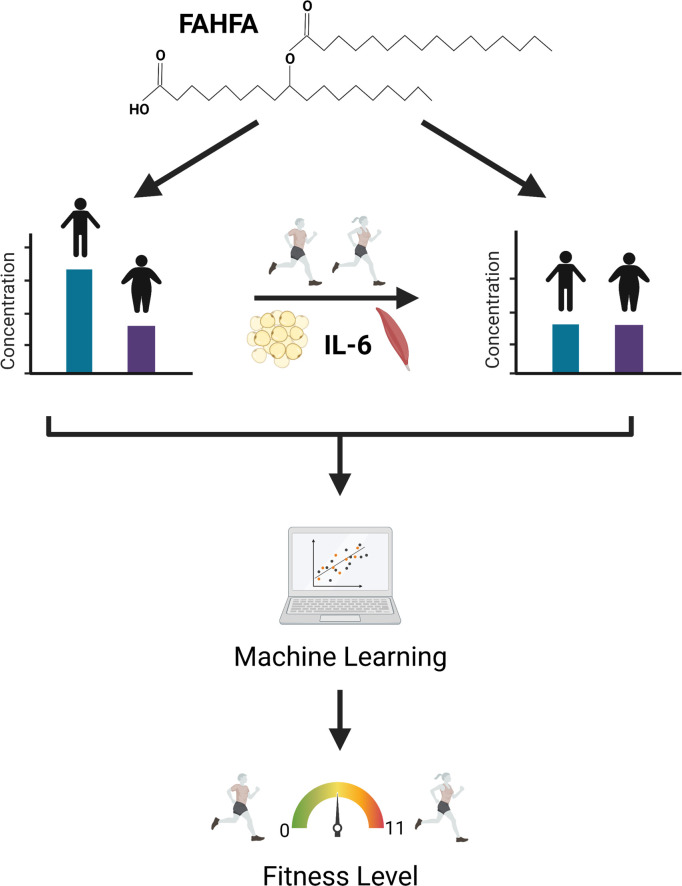

## Introduction

The obesity epidemic and its associated cardiometabolic complications is an acknowledged public health crisis worldwide (1, 2). Obesity, however, does not necessarily equate to obesity-related complications, as observed with metabolically healthy obese (MHO) participants with high BMI (1, 2). Indeed, in MHO patients (prevalence estimate of 7%–13% of adults with obesity), high BMI exists in the absence of any metabolic syndrome components and the presence of low HOMA-IR (1, 2). Additionally, higher cardiovascular fitness, related to higher volumes of physical activity, provides multiple benefits, even after adjusting for BMI, including lower mortality rates, incidence of prediabetes, or incidence of diabetes (3–10). Studies comparing the differences between MHO and metabolically unhealthy obese (MUO) groups focus on identifying differential mortality and morbidity risks and the stability of the MHO state over time (2, 11, 12). Interestingly, 30%–50% of MHO convert to MUO within 4–20 years (11).

Exercise continues to be a highly effective lifestyle intervention for metabolic dysfunction that decreases the risk of more than 20 chronic diseases (13–15). Exercise training improves amino acid and fatty acid profiles over a period of moderate intensity training — adaptations that resemble the metabolic differences between MHO and MUO groups (16–18). Additionally, exercise has an antiinflammatory effect in both acute and chronic settings, contributing to the improvement of insulin sensitivity and other cardiometabolic factors in MUO groups whose members undergo exercise training (13, 19–22). While differences between MHO and MUO groups are evident in the absence of lifestyle intervention, it is plausible that training may protect against transition to MUO. The modulating effect of exercise on insulin sensitivity makes it an important context to study the differential impact of fitness versus BMI on the metabolome. However, the effect of exercise on metabolism has largely been constrained to examining normal-weight trained (NWT) runners or overweight/obese subjects trained from a sedentary state, whereas the population of overweight/obese trained (OWT) runners remains understudied. This group is a relevant demographic since they embody a high-fitness yet obese state whose study may reveal novel physiological adaptation to exercise (2, 6, 16, 23). Furthermore, numerous studies in populations within the normal weight range have interrogated the metabolic responses to acute aerobic exercise. However, nearly all metabolic profiling studies of overweight populations have focused on the sedentary state, or the impact of prolonged exercise interventions and weight loss.

Genomics and epigenomics platforms have uncovered mechanistic drivers at the interface of lifestyle, environmental influences, and metabolic outcomes (24). Metabolomics technologies have also revealed prospective metabolic drivers of obesity pathogenesis and cardiometabolic complications (25). Metabolomics consists of untargeted and targeted approaches that focus on semiquantitative or quantitative measurements, respectively. Targeted metabolomics provides absolute quantities of metabolites; however, only a subset of predefined and validated metabolites are under investigation for formal quantification. On the other hand, mass spectrometry–based (MS-based) untargeted metabolomics surveys global metabolic shifts among samples by measuring the fluctuations of multiple chemical feature abundances detected as *m/z* signals (26, 27). Though untargeted pipelines also capture a large number of artifactual signals, an everimproving toolbox supports chemical validation that allows data set curation and, ultimately, selection of signals for formal validation and quantification (28–33). Therefore, untargeted metabolomics provides a unique opportunity to discover new biomarkers (34–37).

While metabolomics tools have been applied to measure the differences between MHO and MUO, or untrained versus trained groups, here we make the comparison between NWT and OWT groups regarding metabolome differences in the resting state and in response to acute aerobic exercise (16, 20, 38–41). To increase the metabolome coverage that spans polar and nonpolar metabolites, liquid chromatography (LC) methods can be combined. Using serum samples from NWT and OWT participants before and after acute aerobic exercise, we utilized hydrophilic interaction chromatography with negative electrospray ionization (HILIC-ESI[–] MS/MS) and reverse phase (RP) chromatography with positive electrospray ionization (RP-ESI[+] MS/MS) to profile serum metabolites (42). NWT and OWT populations were matched for age, sex, fitness level, and the absence of clinical insulin resistance. Our findings indicate acute aerobic exercise unmasks a latent metabolome in NWT versus OWT participants, supporting an approach that potentially increases the resolving power in the prediction of clinical outcomes, compared with the analysis of samples collected at baseline. Finally, this work also provides a convergent workflow that leverages the complementary strengths of untargeted and targeted metabolomics pipelines.

## Results

### Participant characteristics and study design.

**Figure 1:**
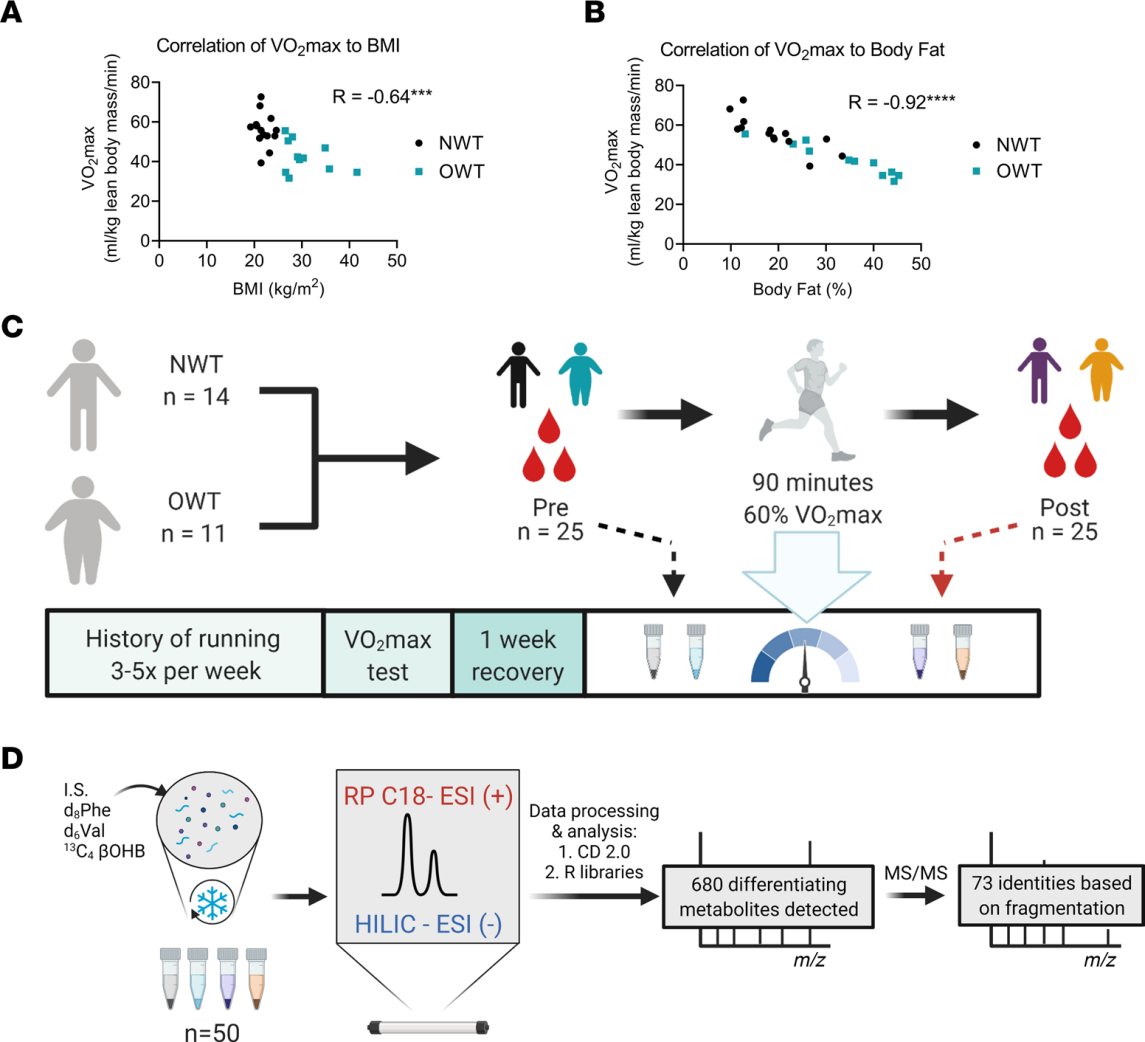
Study design and untargeted metabolomics analytical pipeline. (**A** and **B**) Scatter plots of maximal oxygen consumption (VO_2_max) against BMI and percent body fat. Strength of correlation expressed as Pearson correlation coefficient (*R*). *n =* 25. (**C**) Schematic of study design and sample collection time points. (**D**) Schematic of analytical pipeline for data acquisition. ***P < 0.001, ****P < 0.0001. NWT, normal weight trained runners; OWT, overweight/obese trained group; IS, internal standard; Phe, phenylalanine; Val, valine; βOHB, β-hydroxybutyrate; RP, reverse phase chromatography; ESI, electrospray ionization; (+), positive mode; (–), negative mode; HILIC, hydrophilic interaction chromatography; CD 2.0, Compound Discoverer version 2.0; MS/MS, tandem mass spectrometry.

We enrolled normal-weight and overweight/obese participants with a self-reported history of running 3–5 sessions per week for a minimum of 30 minutes. Trained, rather than sedentary, participants were studied to limit the effects of a deconditioned acute stress response. The cohort consisted of 25 runners (OWT, *n =* 11; NWT, *n =* 14; Table 1). The 2 groups differ by BMI (kg/m^2^, 30.9 ± 0.4 versus 22.1 ± 1.5; *P <* 0.01) and body fat (body fat percentage, 34.0 ± 1.9 versus 19.1 ± 3.1; *P <* 0.01). However, the differences in covariates, such as age, sex distribution, and maximum oxygen consumption (VO_2_max) (mL/kg lean body mass/min) were not significantly different between the groups as determined by 2-tailed *t* test. Though HOMA-IR was higher in the OWT group than the NWT group (1.6 ± 0.1 versus 0.9 ± 0.2, *P =* 0.01), the absolute value of HOMA-IR in the OWT group remained within the range for normal participants (43). Due to absence of cardiometabolic disease, OWT participants met basic criteria defining MHO (2). Correlation analysis with VO_2_max revealed a moderately strong negative relationship between VO_2_max and BMI (*R* = –0.64, *P <* 0.001; Figure 1A) and a stronger relationship between VO_2_max and percent body fat (*R* = –0.92, *P <* 0.0001; Figure 1B). For this study, VO_2_max measures were normalized to lean body mass, which did not significantly differ between groups. These results indicate that fitness is negatively associated with excess body fat, even in trained groups.

At least 1 week prior to this acute running intervention study, the maximum oxygen consumption of each participant was assessed (Figure 1C). The study involved a single 90-minute run at 60% of individual VO_2_max, after overnight fast (at least 8 hours). During the run, participants wore a heart rate monitor; study staff directly supervised each participant to ensure they maintained a heart rate consistent with 60% VO_2_max exertion. Blood samples were collected immediately before and after the running bout. In parallel, all serum samples (*n =* 50) were spiked with 2 internal standards (IS; metabolite naturally absent in the serum) whose signal was monitored to ensure method repeatability. All serum samples were extracted and analyzed using an optimized LC-MS metabolomics protocol (Figure 1D and ref. 44). To ensure the highest metabolome coverage, we acquired the data in positive and negative ionization modes using HILIC (HILIC-ESI[–] MS/MS) and RP (RP-ESI[+] MS/MS) methods (42). Data analyses were performed after ensuring the IS AUCs in all analyzed samples were consistent across the batch (relative SD [RSD] < 10%). Our untargeted workflow removes redundancies that would hinder identification of metabolites of interest in complex LC-MS signal matrices obtained for biological samples. After sample processing, positive and negative mode data sets were merged, and 680 putative metabolites in total were found to be significantly altered relative to preexercise or NWT participants (adjusted *P* < 0.05, log_2_ fold change > 1). We then used targeted LC-MS analyses to formally identify 73 of those metabolites via MS/MS fragmentation that matched with The Human Metabolome Database (HMDB), Metlin, or mzCloud databases (45–51) (Supplemental Tables 1–4 indicate level of confidence for identifications; supplemental material available online with this article; https://doi.org/10.1172/jci.insight.158037DS1).

### Exercise reveals latent differences in the serum metabolome of trained runners.

**Figure 2:**
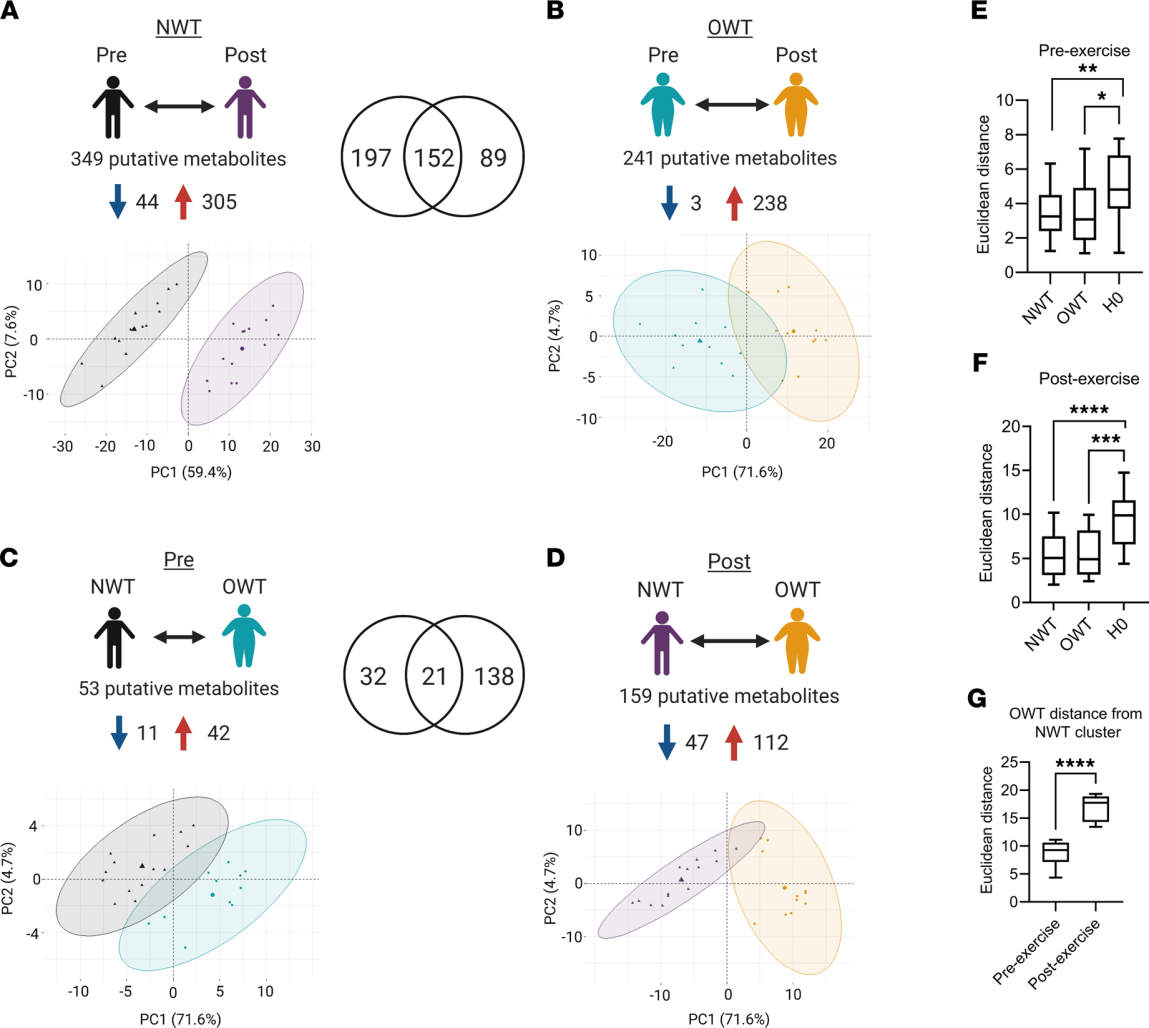
Summary of untargeted metabolomics differential analysis. (**A**–**D**) Metabolic profile differential analysis summary and PCA for 4 group comparisons: exercise effect in NWT (**A**); exercise effect in OWT (**B**); BMI effect at baseline serum conditions (**C**); BMI effect immediately after running (**D**). Blue arrow, decreasing abundance after exercise/OWT; red arrow, increasing abundance after exercise/OWT. Venn diagrams represent overlapping *m/z* and retention time pairs in NWT versus OWT and pre versus post analyses. Percentages on PCA axes represent fraction of explained variance captured by first 2 principal components (Dim1, Dim2). Points inside PCA represent individual samples. Spheres represent normal distribution of group clusters, added after unsupervised PCA analysis in R (FactoMineR and Factoextra packages). (**E** and **F**) Euclidean distance of individual NWT (Dim1, Dim2) and OWT (Dim1, Dim2) from intragroup centroids compared with distance of all individuals from center of all points (denoted H0, as the null hypothesis) for preexercise metabolic profile (**E**) and postexercise metabolic profile (**F**). (**G**) Euclidean distance of OWT individuals (Dim1, Dim2) from center of NWT cluster before and after exercise. *P ≤ 0.05; **P ≤ 0.01; ***P ≤ 0.001; ****P ≤ 0.0001, by Student’s *t* test. Data represent mean ± SEM.

We performed 4 different comparisons: (a) NWT group, before versus after exercise; (b) OWT group, before versus after exercise; (c) before exercise, NWT versus OWT groups; (d) after exercise, NWT versus OWT groups. After correcting for multiple testing by FDR, exercise impacted the abundance of 349 and 241 metabolites in NWT or OWT groups, respectively (Figure 2, A and B, and Supplemental Tables 1 and 2). On the other hand, comparison of metabolome between NWT and OWT groups either before or after a running bout showed that BMI influenced only 53 and 159 differentiating metabolites, respectively (Figure 2, C and D, and Supplemental Tables 3 and 4). To determine the effect of exercise versus BMI, Principal Component Analysis (PCA) was performed using log_10_-transformed abundance of metabolites that significantly differed after correcting for multiple testing. Metabolomics shifts in LC-MS serum profile, and thus PCA group separations, were stronger in NWT or OWT groups where exercise was the studied factor (Figure 2, A and B). The impact of BMI on preexercise serum metabolome was less easily separated by PCA, and this may suggest latent differences in serum metabolome between BMI groups not evident in the resting state (Figure 2, C and D). Euclidean distance was calculated to determine the extent of distinct clustering. For the preexercise metabolic profile comparing NWT with OWT participants, 2 distinct clusters do indeed describe the PCA results (Figure 2E). However, consistent with the larger number of differentially regulated metabolites between the NWT and OWT groups after exercise, compared with before exercise, acute aerobic exercise caused metabolic profiles between OWT and NWT groups to cluster 97.7% farther apart by PCA (Figure 2F). Additionally, distance of OWT participants from the NWT participants cluster centroid is significantly greater after exercise compared with before exercise (Figure 2G). Together with the larger number of disrupted putative metabolites in exercise-induced profiles (349 and 241 for NWT and OWT groups, respectively), these data indicate stronger impact of acute aerobic exercise than BMI on the serum metabolome of trained runners, with acute aerobic exercise unveiling latent differences between NWT and OWT groups.

### Exercise-induced metabolic profiles correlate with circulating cytokines in OWT participants.

**Figure 3:**
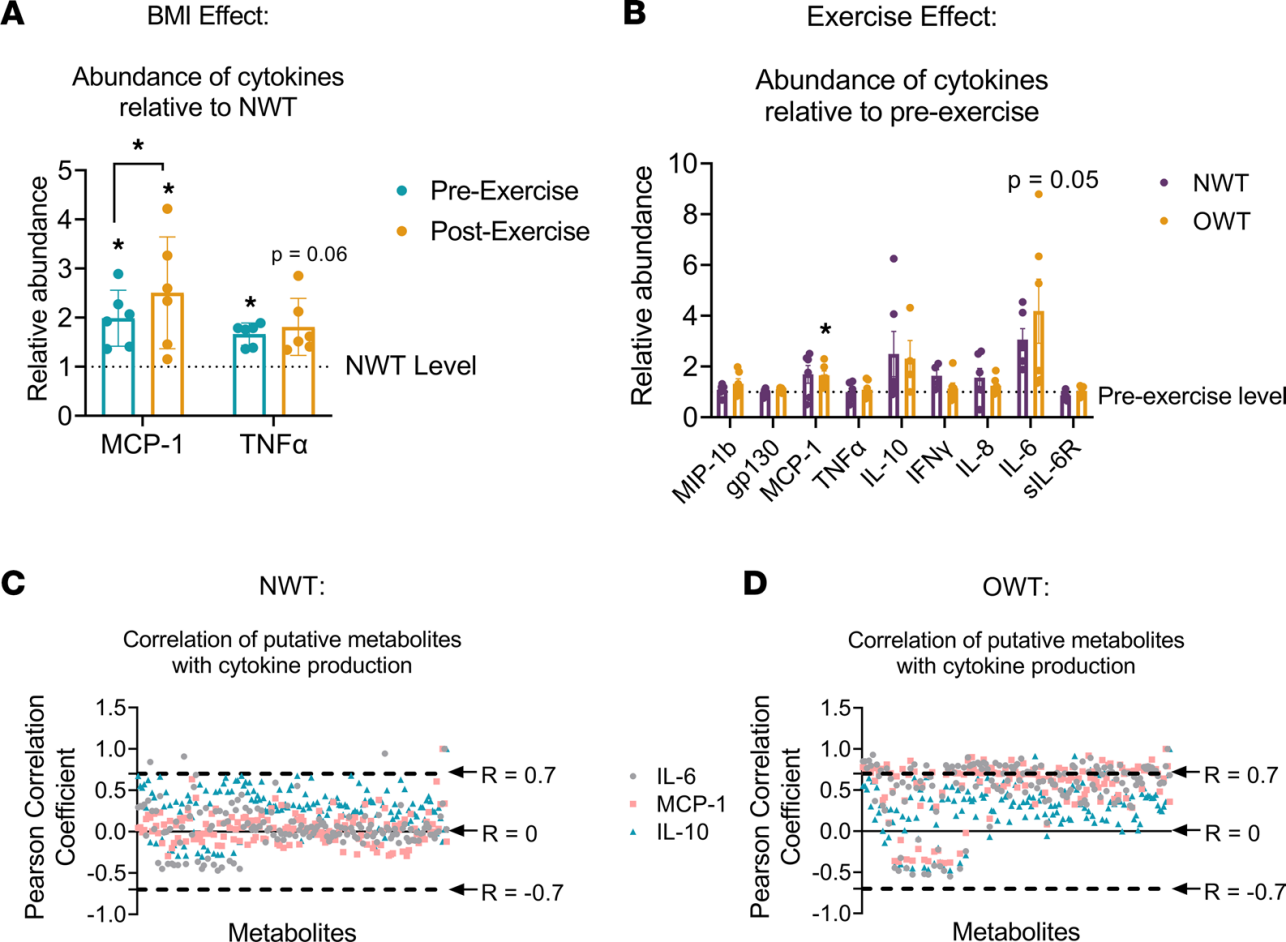
Exercise-induced metabolic profiles correlate with circulating cytokines in OWT. (**A**) Fold change in concentration of circulating MCP-1 and TNF-α in OWT samples relative to NWT; significance symbols without bracket show OWT/NWT comparison, while symbol with bracket shows effect of exercise. (**B**) Fold change in concentration of circulating cytokines relative to before exercise; significance symbol shows pre- to postexercise comparison. (**C** and **D**) Pearson correlation coefficients of 152 intersecting putative metabolites from exercise-related NWT (**C**) and OWT (**D**) profiles against IL-6, MCP-1, and IL-10. Putative metabolites are ordered by *m/z*. *P ≤ 0.05 by Student’s *t* test. *N =* 6 per group. Data represent mean ± SEM.

In addition to serum metabolomics, 12 samples (NWT = 6, OWT = 6) were used to measure cytokines before and after exercise. At baseline, OWT participants had significantly increased serum monocyte chemoattractant protein-1 (MCP-1) (+98.9% ± 23.3, *P =* 0.014) and TNF-α (+66.4% ± 9.3, *P =* 0.012) compared with NWT participants (Figure 3A and Supplemental Table 5). MCP-1 was also elevated after exercise (+150.1% ± 46.4, *P =* 0.016) compared with NWT participants. With exercise, OWT participants had significantly increased circulating MCP-1 (65.5% ± 17.0%, *P =* 0.022) and IL-6 (318.3% ± 126.3%, *P =* 0.05) (Figure 3B and Supplemental Table 5). A correlation analysis between cytokine abundance and intersecting metabolic profiles (152 putative metabolites that did not vary between BMI groups; Figure 2A) revealed that OWT participants exhibited stronger associations with cytokine abundance compared with NWT participants (Figure 3, C and D). Specifically, putative metabolites in the NWT profile group clustered around a Pearson coefficient (*R*) = 0, suggesting low correlation between significantly altered metabolome and cytokine production. Conversely, profile correlations in the OWT participants were much stronger, with most clustering higher around *R* = 0.7 or lower around *R* = –0.5. Statistical significance was determined through Fisher *Z* scores comparing NWT and OWT group Pearson correlation coefficients for each putative metabolite. After adjusting for multiple correction, 8 putative metabolites differentially correlated to IL-6 in OWT participants (*q* = 0.05) (Supplemental Table 6). Together, these data suggest that changes in the metabolome are more directly related to changes in cytokine profiles in OWT participants than in NWT participants.

### Untargeted metabolomics pipeline identifies canonical metabolic profile of exercise.

**Figure 4:**
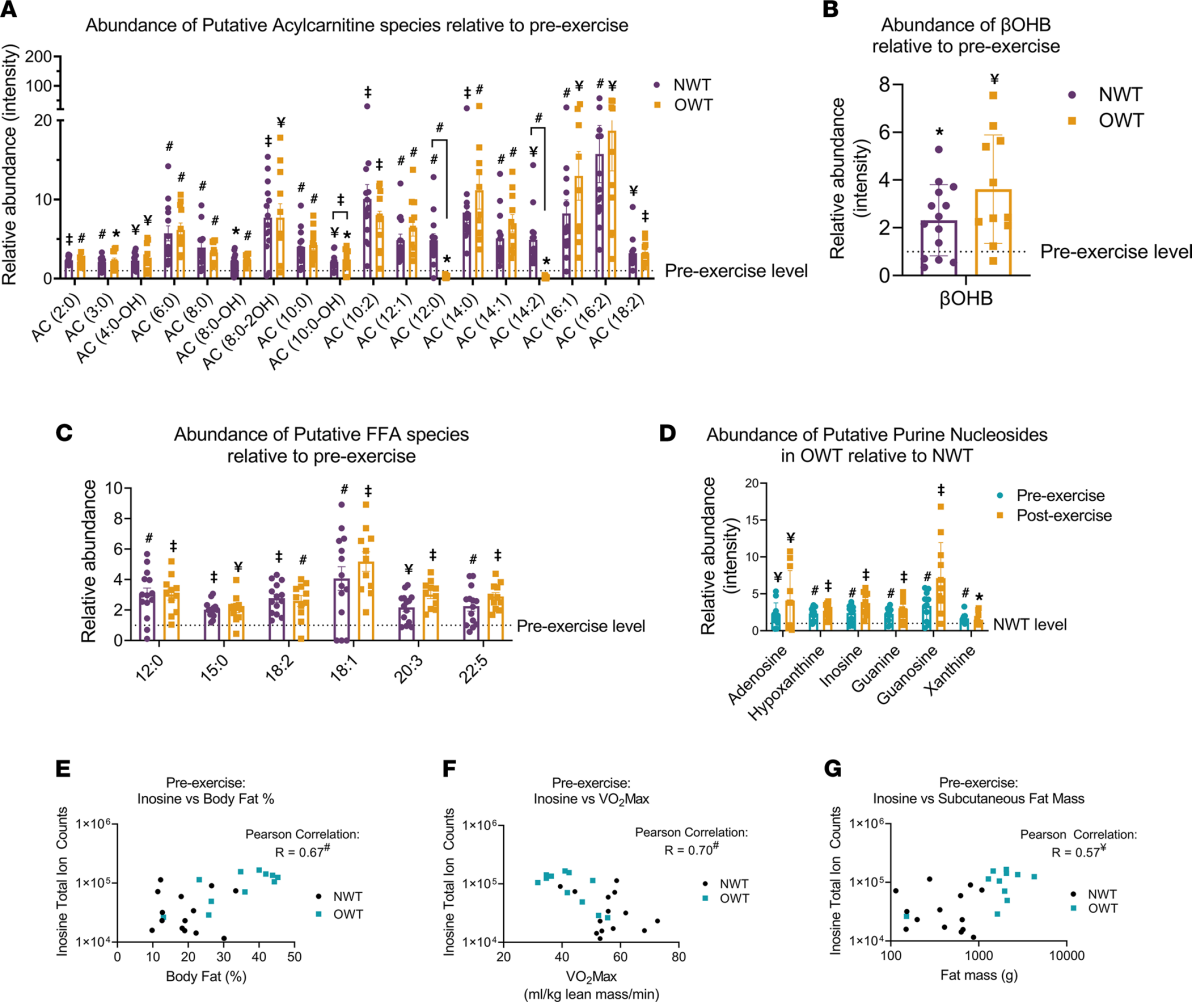
Validation of analytical pipeline. (**A**) Abundances of putative acylcarnitine (AC) species in NWT and OWT groups relative to their respective preexercise abundance. (**B**) Postexercise abundance of βOHB in NWT and OWT groups relative to their respective preexercise abundance, identified by internal standard. (**C**) Abundances of putative free fatty acid (FFA) species in NWT and OWT groups relative to their respective preexercise abundance. Significance symbols (**A**–**C**) denote pre- to postexercise comparison; symbols with brackets denote NWT to OWT comparison. (**D**) Putative purine nucleoside abundance in OWT before and after exercise relative to NWT level; significance symbols indicate OWT to NWT comparison. (**E**–**G**) Scatter plot baseline abundance of inosine for NWT (black circles) and OWT (blue squares) groups against percent body fat (**E**), VO_2_max (**F**), and s.c. fat (**G**). *R* value of correlation pre- and postexercise (NWT + OWT) abundance to study measurements; significance symbols denote comparison to Pearson correlation *R* = 0. Putative species identified by *m/z* and MS/MS fragmentation. *P ≤ 0.05; ^¥^*P ≤* 0.01; ^#^*P ≤* 0.001; ^‡^*P ≤* 0.0001 by Student’s *t* test with Benjamini-Hochberg correction for multiple testing. Data represent mean ± SEM.

Untargeted metabolomics is an important tool for assessing changes in metabolite pools across various groups; however, due to the diversity of methods available for profiling samples, validation of an untargeted method’s workflow for biologically significant features is required. The methods applied in this study revealed several putative metabolite classes known to increase with acute aerobic exercise. Among them were acylcarnitine species, whose identities were supported by comparison of MS/MS fragmentation patterns against the Metlin database (Figure 4A); the ketone body β-hydroxybutyrate (βOHB), which was validated using an authentic internal standard (Figure 4B); and putative free fatty acids (FFA) (Figure 4C). The abundances of these metabolites were significantly increased in both NWT and OWT groups after acute running and confirmed the expected augmentation of adipose tissue lipolysis and hepatic fat oxidation during aerobic exercise. Among these metabolites, no significant differences were observed between NWT and OWT groups after exercise. To validate the observations revealed by the semiquantitative untargeted metabolomics pipeline, FFA species and βOHB were formally quantified using validated shotgun lipidomics and targeted ultraperformance LC–MS/MS (UPLC-MS/MS) approaches, respectively (52, 53) (Supplemental Table 7). These targeted and formally quantitative approaches confirmed those generated through the untargeted metabolomics pipeline: acute aerobic exercise increased βOHB concentrations 2.1- and 3.7-fold in NWT and OWT groups (exercise effect was statistically significant in both groups, but no significant differences between NWT and OWT groups were observed), and FFA concentrations increased after exercise between 1.3- and 4.6-fold in both groups, where exercise effect was statistically significant but where with no significant differences between NWT and OWT groups were observed (Supplemental Figure 1). Similar exercise-associated increases in acetoacetate, an oxidized ketone body redox partner of βOHB, were also observed in both NWT and OWT groups (Supplemental Table 7).

In addition to exercise-engaged lipid metabolites, a small set of metabolites, the purine nucleobases, or nucleosides, including adenosine, hypoxantine, inosine, guanine, guanosine, and xanthine, were significantly increased between 1.6- and 7.1-fold in the OWT group, both at baseline and after exercise (Figure 4D). Identities of these chemical features were confirmed by comparison of MS/MS fragmentation patterns against the Metlin and HMDB databases. These purine metabolites were among the top contributors to differentiating NWT from OWT participants both before and after exercise (Supplemental Table 3, Supplemental Table 4, and Figure 2, C and D). Previous studies identified inosine and hypoxanthine as biomarkers of obesity and cardiometabolic disease (39, 54–56). Our analysis confirmed that inosine abundance shows a strong positive correlation with percent body fat (*R* = 0.67, *P <* 0.0001; Figure 4E) and an inverse correlation with VO_2_max (*R* = 0.70, *P <* 0.0001; Figure 4F). Further analysis of the relationship between putative inosine and fat mass depots revealed a positive association with s.c. fat (*R* = 0.57, *P =* 0.003) (Figure 4G) but not visceral fat (*R* = 0.34, *P =* 0.1). Taken together, the observations generated through our untargeted metabolomics pipeline were supported by targeted approaches and were consistent with the literature, validating our workflow to study the effect of exercise on NWT and OWT participants. Intriguingly, abundances of known exercise-induced metabolites (FFAs, ketone bodies, acylcarnitines) did not vary between NWT and OWT runners, while the serum metabolomics profile differed primarily by purine metabolites associated with obesity.

### Fatty acid esters of hydroxy fatty acids (FAHFAs) decrease in serum with acute exercise in the NWT group.

**Figure 5:**
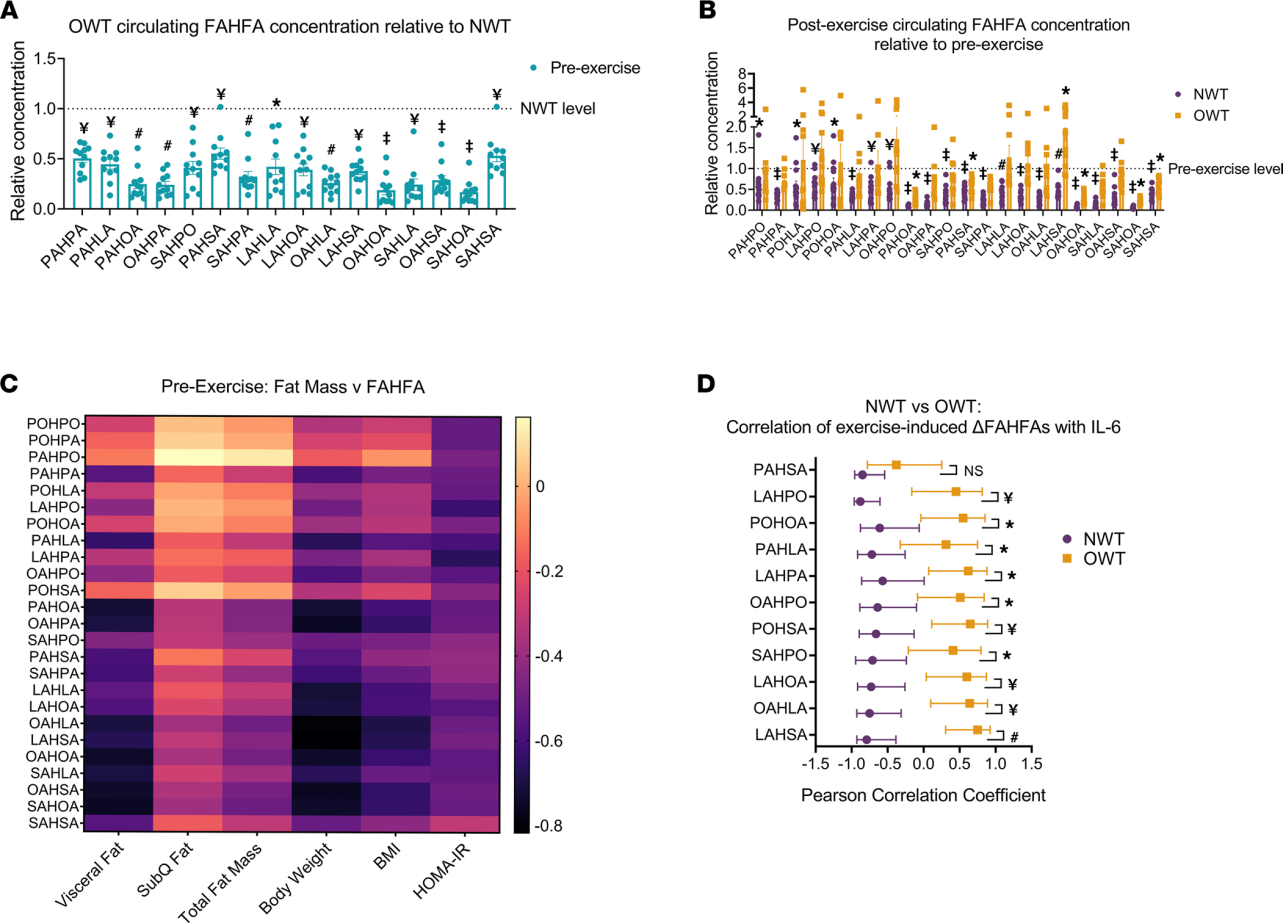
FAHFAs decrease in serum with acute aerobic exercise in NWT but not OWT. (**A**) OWT baseline concentration of circulating FAHFAs relative to NWT; significance symbols indicate OWT/NWT comparison. (**B**) Exercise effect on FAHFA concentration relative to preexercise in NWT and OWT; significance symbols indicate pre- to postexercise comparison. *P ≤ 0.05; ^¥^*P ≤* 0.01; ^#^*P ≤* 0.001; ‡*P ≤* 0.0001 by Student’s *t* test with Benjamini-Hochberg correction for multiple testing. Data represent mean ± SEM. (**C**) Pearson correlation coefficient of fat mass measurements, circulating insulin, and HOMA-IR scores against baseline FAHFA concentrations in NWT and OWT. (**D**) Pearson correlation coefficient of exercise-induced changes in FAHFAs versus IL-6; significance symbols indicate difference in Pearson correlation between NWT and OWT. Significance determined by adjusted *P* value (*q*) after computed Fisher *Z* score. *q ≤ 0.05; ^¥^*q* ≤ 0.01; ^#^*q* ≤ 0.001 by Student’s *t* test. Error bars represent 95% confidence intervals.

Untargeted metabolomics also revealed significant exercise-induced signals that matched the *m/z* of several species from the lipid class FAHFAs. FAHFA lipokines have been associated with antiinflammatory and antidiabetic effects (57, 58). Palmitic acid ester of hydroxy stearic acid (PAHSA) increases in serum and adipose tissue of elderly women after a 4-month training period, though no study has revealed the responses of FAHFAs to acute aerobic exercise in trained runners (59). Given the relevance of FAHFAs to obesity, we directly quantified FAHFA lipid species with a validated shotgun lipidomics approach using NWT and OWT serum samples from pre- and postexercise conditions (60). Consistent with prior reports, baseline concentrations among 16 FAHFA species were diminished 2- to 8-fold in OWT serum compared with NWT (Figure 5A and Supplemental Table 7) (60). Baseline concentrations of PAHSA, commonly observed to be downregulated in obesity, was decreased 1.8-fold in OWT serum relative to NWT. Other FAHFA species, such as stearic acid ester of hydroxy oleic acid (SAHOA, 5.9-fold lower in OWT relative to NWT serum), were even more suppressed in OWT compared with NWT participants (Figure 5A). Strikingly, acute aerobic running decreased the concentrations of 22 of 25 quantified FAHFA species by 34%–94% in the NWT group, while in OWT participants, only 5 of those were decreased, by 40%–79% (Figure 5B). In fact, in the OWT group, 1 FAHFA species, a linoleic acid ester of hydroxy stearic acid (LAHSA), was significantly increased (median increase of 73%) with acute running, while no FAHFA species increased with running in the NWT group. Notably, both the acute running-induced decreases in the NWT group and the dysregulated dynamic responses observed in the OWT group were in contradistinction to the conserved increases in FFA levels after exercise in both NWT and OWT runners that were observed using both untargeted and targeted approaches (Figure 4C and Supplemental Table 7).

The disparate effect of acute aerobic exercise between BMI groups on static FAHFA concentrations suggests that FAHFA turnover during exercise may be modulated by body fat and/or relative insulin resistance. To determine if baseline FAHFA concentrations were related to specific adipose depots, we performed Pearson correlation analysis, which revealed that many species were negatively correlated with visceral fat mass, total body weight, and BMI (Figure 5C) but not s.c. or total body fat mass. Several of the species with the strongest negative associations (e.g., palmitic acid hydroxy ester of oleic acid [PAHOA], oleic acid hydroxy ester of oleic acid [OAHOA], and SAHOA) were those that decreased with exercise in both NWT and OWT groups, despite OWT showing lower circulating concentrations before exercise. As expected, FAHFAs also negatively correlated with HOMA-IR (Figure 5C). Because specific adipose depots have a differential impact on circulating cytokines, we next determined if the observed variations in exercise-induced changes in FAHFA concentrations unveiled latent relationships between BMI and circulating inflammatory cytokine levels. Linear correlation analysis of covariance revealed that numerous FAHFA species showed a strong, inverse relationship with circulating IL-6 abundance in the NWT group (Figure 5D and Supplemental Table 8), while the OWT group showed nominally positive relationships to IL-6. The species exhibiting these relationships are those that decrease with exercise in NWT, but not in OWT. Therefore, while IL-6 was not significantly different between BMI groups (Figure 3B and Supplemental Table 5), this correlation suggests that there are pathways linked to IL-6 and acute aerobic exercise involving FAHFAs that are dynamically regulated by adipose tissue mass. Taken together, integrated, adipose depot–specific, and FAHFA species–specific mechanisms may modulate both FAHFA turnover and cytokine production. Furthermore, exercise-responsive regulation of FAHFAs could depend on selective adipose depots and cytokine action, as several FAHFAs are selectively regulated in OWT, despite depleted abundance at baseline.

### Exercise reveals BMI impact on FAHFA and FFA fold changes in trained runners.

**Figure 6:**
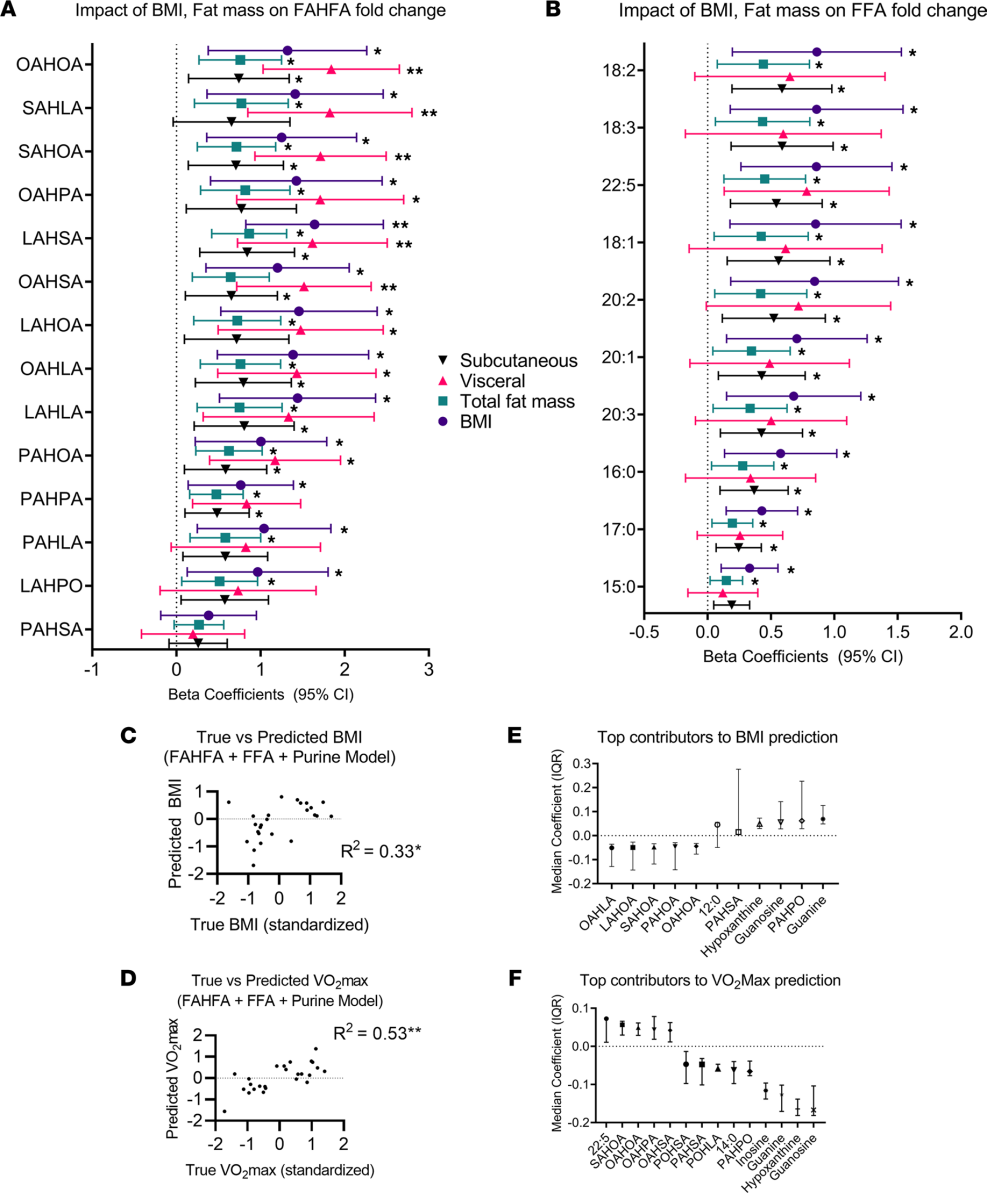
BMI and visceral fat impact FAHFA turnover after acute running in trained groups. (**A** and **B**) Regression coefficients with 95% CI for impact of incremental BMI and fat mass measurements on fold-change (before to after exercise) of FAHFAs (**A**) and FFAs (**B**); significance symbols denote adjusted *P* value of relationship between fat depot and metabolite fold change. (**C**) Scatter plot true BMI versus predicted BMI after 5-fold cross-validation. (**D**) Scatter plot VO_2_max versus predicted after 5-fold cross-validation; significance symbol indicates comparison to Pearson correlation *R*^2^ = 0. (**E** and **F**) Median values with IQR of top positive and top negative coefficients from ridge regression predicting BMI (**E**) and VO_2_max (**F**). Median determined after 5 different models, 1 for each training set split (see Methods for details). *P ≤ 0.05; **P ≤ 0.01 by Student’s *t* test with Benjamini-Hochberg correction for multiple testing.

As described above, we observed an increased number of significantly altered putative metabolites between NWT and OWT groups after exercise and surprising differences in metabolite associations to circulating cytokines (Figure 2, D and G, and Figure 3, B–D). As a result, we postulated that metabolomics signatures could predict anthropometric and/or performance indices. Therefore, we performed regression modeling of intersecting metabolic profiles for OWT and NWT groups to determine if exercise-induced changes in metabolite abundance are associated with BMI. The log_2_ fold-change of absolute quantities of FAHFA and FFA species acquired using formally quantitative shotgun lipidomics methods were individually regressed against BMI (in +10 kg/m^2^ increments), total fat mass (per +10.0 kg), visceral fat mass (per +0.5 kg), and s.c. fat mass (per +1.0 kg). β Coefficients captured the effect of increments on lipid species fold change. Many FAHFA species are influenced by BMI, s.c. fat mass, and visceral fat mass with an FDR < 0.05, while FFA species are impacted by BMI and s.c. fat but not by visceral fat mass (Figure 6, A and B). The FAHFA species whose fold-change with exercise is most impacted by visceral fat mass are OAHOA, SAHLA, and SAHOA — species whose baseline concentrations negatively correlated with visceral fat mass (Figure 5D). Notably, exercise-induced fold-change of PAHSA was not found to be significantly influenced by BMI or visceral fat in this trained population. FFA species most impacted by BMI during exercise were unsaturated 18:2 (linoleic acid) and 18:3 (linolenic acid). Long chain fatty acids, 22:5 (docosapentaenoic acid [DPA]) and 20:3 (eicosatrienoic acid [ETE]) are precursors to eicosanoids, and other lipid mediators that are derived from elongation steps of 18:2 and are also significantly impacted by BMI. Together, these results underscore that BMI and specific fat depots are differentially associated with FAHFA and FFA turnover. Targeted shotgun lipidomics showed modest differences in FFA fold change with exercise between NWT and OWT that were not statistically significant, suggesting that variation in FFA is related to underlying differences in metabolic activity of s.c. adipose depots (Supplemental Figure 1).

To evaluate the ability of combined preexercise FAHFA, FFA, and purine nucleoside abundances to predict BMI and cardiovascular fitness (VO_2_max), we applied a machine learning approach to these data. To prevent model overfitting, we applied regularized regression models (ridge regression), which include a penalty during model fitting to yield sparse models that depend only on the most influential metabolites. Resulting models applying FAHFA + FFA + purine profiles predicted BMI with an average *R*^2^ = 0.33 (explaining 33% of variance in BMI, *P =* 0.03), while prediction for VO_2_max reached *R*^2^ = 0.53 (explaining 53% of the variance in VO_2_max, *P =* 0.002; Figure 6, C and D); these are magnitudes consistent with other metabolomics correlation studies (39). The most significant contributors for BMI and VO_2_max are shown in Figure 6, E and F (all model coefficients can be found in Supplemental Tables 9 and 10). A positive model coefficient indicates a direct relationship to the predicted variable (BMI or VO_2_max), while negative coefficients indicate an inverse relationship with the predicted variable of interest. Interestingly, purines grouped with several FAHFA species, including PAHSA, with a negative relationship to VO_2_max, while the remaining highly ranked FAHFAs had the opposing sign. Among these, palmitic acid ester of hydroxy palmitoleic acid (PAHPO), palmitoleic acid ester of hydroxy linoleic acid (POHLA), and palmitoleic acid ester of hydroxy stearic acid (POHSA) were among the few FAHFAs that did not differ significantly between NWT and OWT at baseline and may be important species for training adaptation independently of BMI (Supplemental Table 7). Notably, PAHSA was not highly ranked for predicting BMI. Oleic acid ester of hydroxy oleic acid (OAHOA), the FAHFA with an exercise-induced fold change that was most influenced by visceral fat mass, also contributed to the prediction of both BMI and VO_2_max (Figure 5D and Figure 6, E and F). SAHOA also contributed to both BMI and VO_2_max, while some FAHFA species selectively contributed to only BMI or VO_2_max prediction. While the significance of fatty acid chain versus hydroxy fatty acid chain is not well understood in the synthesis of FAHFAs, our results show they tend to change to similar degrees with exercise. More study is required to determine how particular FAHFA composition, including regioisomerization, is to physiological impact (61). These data suggest that FAHFAs play a role in the training effect of chronic running and may be in opposition of adenine metabolism–related effects. FAHFAs have been primarily studied in relation to their influence on insulin sensitivity, but this study reveals a novel role in acute aerobic exercise adaptation.

## Discussion

This study recruited an understudied population of OWT participants to assess differences in the serum metabolome compared with NWT counterparts before and immediately following nonexhaustive aerobic exercise. The OWT group shares similarities with MHO, an important demographic, whose fitness may preempt transition to MUO. NWT and OWT groups did not differ in their fitness levels, as determined by VO_2_max, allowing evaluation of BMI and body fat effect on the response to exercise. Notably, BMI was not associated with substantial differences in serum metabolome either before or after acute aerobic exercise in trained OWT or NWT runners. Lipidomics measurements showed exercise-induced depletion of circulating FAHFA lipokines in NWT individuals, including several species that have yet to be studied in depth (Figure 5B). Finally, a machine learning approach also revealed that FAHFAs strongly predict VO_2_max (Figure 6, D and F).

The mechanisms of FAHFA turnover, including acyl chain specificity in FAHFA formation, have not been completely elucidated. Thus, the differential influence of individual FAHFA families is of interest. FAHFAs were first identified by Kahn and colleagues as a class of Glut4-regulated lipids that positively correlated with insulin sensitivity (58). Chronic PAHSA treatment in mice fed a high-fat diet improved glucose uptake and insulin sensitivity in heart, skeletal muscle, and liver and protects against colitis in the gut (62, 63). Efforts to identify regulation of FAHFA synthesis have linked these lipids to the Nrf2-regulated antioxidation pathway (64). While studies to uncover regulation of FAHFA synthesis and hydrolysis are ongoing, carbohydrate response element binding protein–regulated (ChREBP-regulated) de novo lipogenesis and the Nrf2 antioxidant system in adipose tissue may play important roles (58, 64). Nrf2 is activated during exercise, increasing activity of antioxidant defense pathways (65, 66), and suggests a possible role of FAHFAs in response to training. Brezinova et al. identified increases in baseline PAHSA synthesis after 4 months of training in sedentary, elderly women (59). Further research into the role of FAHFAs in training are necessary and may reveal novel adipose tissue physiology in exercise adaptation.

Exercise produced similar yet distinct metabolic profiles between NWT and OWT groups that differentially associated with circulating IL-6, further suggesting an underlying influence of BMI on the physiological effect of acute running. During exercise, IL-6 mediates crosstalk between skeletal muscle and target organs, including adipose tissue, possibly stimulating lipolysis and mobilization of nonesterified fatty acids (14, 67–70). IL-6 can also be induced by Nrf2 under conditions of oxidative stress. Previous study of the association between IL-6 production and lipid metabolite abundance in lean, male runners saw only a minor relationship after endurance running (71). Thus, our study is the first to our knowledge to show BMI-related shifts in metabolites that present a stronger relationship to IL-6 and MCP-1 production and may represent an important difference in exercise physiology due to excess body weight. Circulating metabolite and cytokine abundance is limited in its ability to reveal directionality. Correlations suggest a possible relationship between IL-6 and FAHFA clearance from circulation in NWT participants. Dynamic turnover of circulating FAHFAs could be attributable to numerous mechanisms that are not mutually exclusive, including synthesis, hydrolytic catabolism, or uptake/release within tissues. Our lipidomics platform measures FAHFAs composed of 16:0, 16:1, 18:0, 18:1, and 18:2, with complement hydroxy fatty acids, which decreased during exercise. Depletion of FAHFAs during exercise may be due to hydrolysis to provide fatty acids for oxidation during a long running bout (60). However, FAHFAs may also regulate glucose transport through activation of GPR120; thus, their downregulation during exercise may be due to metabolic switch from glucose to fatty acid oxidation (57, 58). However, FAHFA roles in skeletal muscle remain largely unknown. Our results are consistent with the notion of metabolic crosstalk between specific adipose tissue depots and skeletal muscle. Further study of FAHFAs in trained contexts is needed to elucidate this relationship.

Together with FAHFAs, purine nucleosides contributed to VO_2_max prediction by machine learning models. Purines, including putative inosine and hypoxanthine, were among the top metabolites contributing to variation between NWT and OWT participants before and after exercise and were highly associated with percent body fat. Inosine showed fold-changes of 2.4 before exercise and 3.7 after exercise in OWT over NWT participants. Elevation in adenine catabolism products has been reported in metabolomics analyses of pathologies associated with obesity, and its levels improve with exercise training (55, 72–75). A recent multiomics analysis identified a downstream metabolite of purine nucleotides, uric acid, as highly associated to BMI in participants with an abnormal metabolome (39). Exercise training of *db/db* mice showed restoration of uric acid and its intermediates in skeletal muscle (74). Increasing levels of hypoxanthine have also been observed in human adipose tissue under hypoxic conditions (56). These results may reflect ongoing risk for obesity-related pathologies in OWT (14, 67–71, 76–78).

Exercise is a “formidable regulator of insulin sensitivity and overall systemic metabolism” (14). Acute and chronic effects of exercise force adaptation in several systems, including adipose tissue, skeletal muscle, and the liver. For this reason, exercise continues to be the most effective intervention for metabolic diseases, such as type 2 diabetes and cardiovascular disease, and could be an important strategy in preventing MHO to MUO conversion. This study showed that FAHFAs and purine nucleosides significantly contributed to variation in VO_2_max after normalizing for lean body mass. Intriguingly, FAHFAs were negatively associated with visceral fat mass, while inosine was positively associated with s.c. fat. These relationships may indicate competing metabolic impacts from specific adipose depots that influence overall metabolic health. Future studies to uncover the role of FAHFAs in both acute and chronic exercise may provide insight into adipose tissue remodeling in exercise and offer a node for therapeutic intervention.

### Limitations.

This study has several important considerations. We report the metabolomics shift in serum of well-trained participants with normal and high BMI. Previous studies have demonstrated that BMI incompletely characterizes metabolic health (39). Some participants within the OWT group had very low body fat, and their exercise-induced changes were minimal for the identified metabolic profile (Figure 2B). This cross-sectional study sought participants with an established exercise habit and did not acquire further details on training history, diet, or body composition prior to training. These factors need to be considered in future human studies. Due to the small sample size, additional studies of FAHFAs in both untrained and trained participants are required to demonstrate reproducibility of the relationships among FAHFAs, cardiovascular fitness, and long-term health outcomes.

## Methods

### Study participant details.

We recruited OWT (*n =* 11) and NWT (*n =* 14) participants who self-reported aerobic exercise (3–5 sessions/week) from the Twin Cities metro area (Minneapolis, Minnesota, USA) between July 2014 and April 2017. We preferentially recruited participants from recent running events, to ensure that they are capable of completing a prolonged (90-minute) run. Inclusion criteria included: (a) age between 18 and 40 years and (b) regular aerobic exercise, preferably running, at least 3 to 5 sessions/week. Participants with (a) self-reported clinically significant medical issues (e.g., diabetes, cardiovascular disease, uncontrolled pulmonary disease), (b) abnormal electrocardiogram indicating cardiac disease (study electrocardiogram performed), or (c) current pregnancy (screening pregnancy test performed) were excluded. Participants were recruited with the goal to achieve similarity in age and sex between the 2 groups.

### Assessment of insulin sensitivity.

Blood was drawn after an 8-hour fast to measure insulin and glucose levels to calculate insulin sensitivity, as estimated by HOMA-IR (fasting serum insulin [μU/mL] × fasting glucose [mmol/L])/22.5) (43, 79).

### Body composition.

Body composition was measured by dual-energy X-ray absorptiometry (DXA). Body composition was measured by a DXA scanner, the GE Healthcare Lunar iDXA (GE Healthcare Lunar with enCORE software version 16.2).

### VO_2_ max.

Subjects were instructed to refrain from intentional exercise for 72 hours and to eat a light snack 2–3 hours before testing. VO_2_max was evaluated by indirect calorimetry using 1 of 2 metabolic carts, either at the Human Performance Teaching Laboratory (Ultima Medgraphics CPX-D, Medical Graphics Corporation, St. Paul, Minnesota, USA) or Masonic Clinical Research Unit (ParvoMedics TrueOne 2400 – OUSW 4.3.4 [20160202], Sandy, Utah, USA). Fitness was quantified by VO_2_max, normalized for lean mass.

### Acute aerobic exercise intervention.

The acute aerobic exercise intervention was scheduled at least 1 week after the VO_2_max testing to minimize influence of the strenuous exercise from the VO_2_max test. Subjects were instructed to avoid intentional exercise for 2 days before the second visit and arrive after an overnight fast (at least 8 hours). The exercise bout was a prolonged run designed to promote fat oxidation. The heart rate reserve (HRR) was calculated from the subject’s resting heart rate and maximum heart rate from the VO_2_max testing. For the supervised exercise bout, all subjects ran for 90 minutes on a treadmill. Each subject’s run pace was initially selected by adjusting the speed and incline that achieved 60% HRR during the VO_2_max, to keep all participants running at 0% grade. Heart rate was monitored during the entire run by study staff to maintain proper running intensity with the Polar heart rate monitor and the Polar Beat Multi-Sport Fitness Tracker smartphone app (Polar Electro Inc.). If a heart rate fluctuated by more than 5% of the HRR, study staff adjusted the treadmill speed in 0.2 mph increments until the target heart rate was maintained. Subjects were offered free access to water during their exercise bout. Plasma samples were collected before and after the acute aerobic exercise intervention.

### Cytokine analysis.

For a subset of the participants (*n =* 6/group), IFN-γ, MCP-1, TNF-α, IL-6, IL-8, and IL-10 were measured before and after acute aerobic exercise. Samples were tested by the Cytokine Reference Laboratory (CRL, University of Minnesota). Samples were analyzed using the Luminex platform and done as a High-Sensitivity multi-plex assay. The magnetic bead set (catalog LHSCM000; lot no. P127677) was purchased from R&D Systems. Samples were assayed according to manufacturer’s instructions. Fluorescent color-coded beads coated with a specific capture antibody were added to each sample. After incubation, and washing, biotinylated detection antibody was added followed by phycoerythrin-conjugated (PE-conjugated) streptavidin. The beads were read on a Luminex instrument (Bioplex 200), which is a dual-laser fluidics based instrument. One laser determines the analyte being detected via the color coding; the other measures the magnitude of the PE signal from the detection antibody, which is proportional to the amount of analyte bound to the bead. Samples were tested using singlet testing, and values were interpolated from 5-parameter fitted standard curves.

### Sample preparation for untargeted metabolomics study.

Samples were stored at –80°C and extracted just prior to the analysis using the protocol from Ivanisevic et al. with modifications (42). For quality control, serum was spiked with IS of d_8_-Phe, d_8_-Val, and ^13^C_4_-βOHB (10 μM each in final extract), normally absent in the serum and ion intensity monitored along the batch analysis. Briefly, 20 μL of serum was extracted with 80 μL of cold (–20°C) MeOH/AcN (1:1, v/v) solution and submitted to the vortexing and bath sonication (10 minutes). The samples were incubated at –20°C for 1 hour, spun to remove proteins, transferred to a fresh tube, evaporated, and reconstituted in 100 μL of 1:1 AcN/H_2_O (1:1, v/v). Prepared samples were vortexed, sonicated (10 minutes), centrifuged (10 minutes, 20,000*g* at 20°C), and analyzed.

### Untargeted LC-MS metabolomics analysis.

The untargeted analysis was performed on Dionex Ultimate 3000 RSLC liquid chromatograph hyphenated with Thermo Q-Exactive Plus mass spectrometer equipped with heated ESI source. Samples were analyzed using positive or negative MS mode using either RP or HILIC-LC separation conditions, respectively. RP separation was carried out using positive mode on Atlantis T3 (150 × 1 mm, 3 μm) column using mobile phase A (water + 0.1% formic acid), and mobile phase B (AcN + 0.1% formic acid) with binary gradient of 5%–95% B for 50 minutes, 95%–5% B for 7 minutes, and 5% B for 13 minutes. HILIC separation was performed using negative mode on Luna Aminopropyl (150 × 1 mm, 3 μm) using mobile phase A, 10 mM ammonium acetate/10 mM ammonium hydroxide in 95% water, and mobile phase B, 10 mM ammonium acetate/10 mM ammonium hydroxide in 95% water, 5% AcN. Separation was performed using binary gradient of 100% to 0% B for 50 minutes, 0% B for 7 minutes, and 100% B for 13 minutes. All separations were performed at flow rate 50 μL/min and column temperature at 30°C. HILIC analyses used an injection volume of 4 μL, and RP used an injection volume of 1 μL. The mass spectrometer operated in negative full scan (FS) mode (*m/z* 68–1020) was used with optimized heated electrospray ionization (HESI) source conditions: auxiliary gas 10, sweep gas 1, sheath gas flow at 35 (arbitrary unit), spray voltage –3 kV, capillary temperature 275°C, S-lens RF 50, and auxiliary gas temperature 150°C. For positive FS mode (*m/z* 68–1020), the optimal conditions were: auxiliary gas 10, sweep gas 3, sheath gas flow at 30 (arbitrary unit), spray voltage 4 kV, capillary temperature 350°C, S-lens RF 50, and auxiliary gas temperature 120°C. The AGC target was set at 1 × 10^6^ ions and resolution at 70,000. Samples within the sequence were injected in randomized order to minimize the possibility of column carryover. The signals of d_8_-Phe, d_8_-Val, and ^13^C_4_-βOHB were extracted from all analyzed samples, and the RSD of the area among all samples was below acceptable 10%.

To identify selected metabolites, MS/MS analysis was performed on a Thermo Vanquish liquid chromatograph, with all other LC-HESI conditions unchanged. Data were first acquired in FS to assess retention time drift between the LC systems, revealing a 10-minute global retention time shift. Features of interest were targeted by parallel reaction monitoring (PRM) using a 1.0 *m/z* window and 5-minute retention time window for each signal. For targeted MS/MS of ions, the following MS/MS settings were applied: resolution at 35,000; AGC target of 2 × 10^5^, and maximum IT of 100 ms. Normalized collision energy was applied in steps from 20% to 40%, to 100%.

### Data processing.

Untargeted metabolomics RAW files were treated using Compound Discoverer 2.0 software. Positive and negative mode data processing used the following parameters: mass tolerance, 5 ppm; maximum retention time drift, 4 minutes using the adaptive curve algorithm, minimum scans per peak totaled 5; and maximum peak width (full-width-half-height), 0.5 minutes. We excluded background signals using parameter setting Sample/Blank signal ratio > 3 and merged chemical features corresponding to isotopes and adducts into 1 putative metabolite (29). Results from negative- and positive-mode data were analyzed separately. Differential abundance analysis was performed to analyze the effect of (a) BMI or (b) acute exercise. Peaks with log_2_ fold change > 1 and *P* < 0.05 were selected and evaluated using Compound Discoverer 2.0 visualization tools for quality of spectra, peak picking, and area integration. Poorly integrated peaks of interest were manually integrated in Thermo Quan Browser. Further negative and positive results were manually merged. Metabolites of interest were targeted using MS/MS, and the identification was based on the MS/MS spectra available in publicly accessible HMDB, Metlin, or mzCloud databases (45–51).

### Quantitation of FAHFA and FFA.

Quantification of FAHFA and FFA was carried out using the validated internal standard addition methods. FAHFA and FFA species were identified and quantified through multidimensional MS-shotgun lipidomics, as previously described (53, 60). Briefly, serum were spiked with an appropriate amount of IS d_4_-16:0 FFA or 12-PAHSA-d_4_, and lipids were extracted using modified Bligh and Dyer protocol. FAHFA samples underwent solid-phase extraction with a HyperSep silica SPE cartridge at room temperature. After conditioning the SPE cartridge with 15 mL hexane, previously dried-down samples were reconstituted in 200 μL chloroform and loaded into the cartridge. Neutral lipids were eluted with 15 mL of 5% ethyl acetate in hexane, followed by elution of FAHFAs with 10 mL of ethyl acetate. Eluent was dried under N_2_ stream.

FAHFA and FFA extracts were derivatized with *N*-[4-(Aminomethyl)phenyl]pyridinium (AMPP) agent, as previously described (53, 60). Briefly, 10 μL of ice-cold acetonitrile/*N,N*-dimethylformamide (4:1, v/v) was added to lipid extracts. Then, 10 μL of ice-cold 640 mM (3-[dimethylamino]propyl)-ethylcabodiimide hydrochloride aqueous solution was added. After vortexing, 10 μL of ice-cold 10 mM *N*-hydroxybenzotriazole and 10 μL of 30 mM AMPP (dissolved in acetonitrile) were added. Each tube was vortexed, filled with N_2_, capped, and incubated at 68°C for 1.5 hours. Derivatives were then extracted with 4.5 mL of CHCl_3_/MeOH/H_2_O (1:1:1, v/v/v). The bottom layer was collected, dried under N_2_ stream, and resuspended in 50 μL of CHCl_3_/MeOH (1:1, v/v).

Derivatized extracts were infused in TSQ triple quadrupole mass spectrometer (Thermo Fisher Scientific) equipped with an automated nanospray device (Trivers Nanomate, Advion Biosciences). Identification and quantification of derivatized FFA was performed using selected precursor ion mode scans using in-house automated software. FAHFA identification and quantitation was performed in product-ion analysis. Optimized collision-induced dissociation was also used for neutral-loss scanning (60). Data processing was performed as previously described (80).

### Quantification of Ketone bodies.

Ketone bodies were quantified using a fully validated UPLC-MS/MS method as described elsewhere (52). Briefly, serum samples were extracted using cold ACN/MeOH (1:1 v/v) with addition of d_4_-βOHB and [^13^C_4_]-acetoacetate (AcAc) as internal standards; it was then vortexed and centrifuged in 4°C at 15,000*g* for 10 minutes. The LC-MS/MS method can be found in previously published protocol (52). Absolute concentration was determined through Thermo Quan Browser.

### Statistics.

Descriptive data are expressed as mean ± SEM for continuous measures and *n* (%) for categorical measures (Table 1). Comparisons between the NWT and OWT groups were performed using paired 2-tailed *t* test. Statistical significance was defined as *P* ≤ 0.05 due to the sample size and exploratory nature of this study. All statistical analyses of metabolite profile used Benjamini-Hochberg to adjust for multiple testing across metabolites (81). Cytokine analyses were performed using SAS 9.3 (SAS Institute). PCA used log_10_-transformed raw intensities and R packages FactoMineR and Factoextra.

The correlation between metabolic profiles and cytokines IL-6 and MCP-1 was computed for each metabolite individually using the Pearson correlation in the R base package, based on log-transformed raw intensities and cytokine abundance. The significance of differential correlations were calculated using the Fisher *Z* score, corrected for multiple testing, and graphed to show 95% CI.

The relationships between exercise-related change in metabolite levels (difference in log_2_ concentrations between post- and preexercise results) and preexercise BMI, total fat mass, visceral fat mass, and s.c. fat mass were examined using linear models of metabolite changes, in univariate models and in models including sex and age covariates. Effects are reported as model coefficients with 95% CI after correction for multiple testing.

For predictive models for BMI and VO_2_max, ridge regression was used to build models using glmnet library in R. LASSO was also tested but was outperformed by ridge in terms of model generalizability; thus, results from ridge are reported here. Due to small sample size, 5 unique models were generated and are summarized. Samples were randomly sampled without replacement for 5 unique training and testing sets of combined NWT and OWT (80/20 split), and the sampling was constrained to maintain the participant sex distribution in each split to remove sex differences as a confounding factor. For each training/test set split, the lambda parameter was tuned based on the training set only using 10-fold cross-validation. The optimal lambda was chosen on the basis of the training set cross-validation performance, and the resulting model was then used to predict BMI or VO_2_max on the test set. All correlation analyses with model predictions are based on test set predicted values. Model variables included scaled concentrations of FAHFA and FFA species, scaled intensities for putative purine species, scaled age, and sex. Performance was evaluated based on the Pearson correlation coefficient between the test set predicted values and actual values. Additional models were trained with the inclusion of sex and age; however, these did not significantly improve model performance. Optimal lambda values and variable coefficients for each training set can be found in Supplemental Tables 9 and 10. Data were plotted and statistical analysis was performed on Prism (GraphPad) v9.0 and in R Studio (v3.6.3) unless otherwise noted. Numbers of observations, assessments of normal distributions, and statistical tests applied are provided in the figure legends.

### Study approval.

The University of Minnesota’s IRB approved the study protocol and methods. All participants provided written informed consent before study participation. The study is registered at ClinicalTrials.gov, accession NCT02150889.

## Author contributions

ABN, PP, CCH, CLM, LSC, and PAC contributed conception and design of the experiments. ABN, PP, JRG, DBS, MP, XH, and ME contributed collection, analysis, and interpretation of data. ABN, PP, LSC, and PAC contributed drafting and revising the manuscript. All authors have read and approved the final submission.

## Supplementary Material

Supplemental data

ICMJE disclosure forms

Supplemental table 1

Supplemental table 2

Supplemental table 3

Supplemental table 4

Supplemental table 5

Supplemental table 6

Supplemental table 7

Supplemental table 8

Supplemental table 9

Supplemental table 10

## Figures and Tables

**Table 1:**
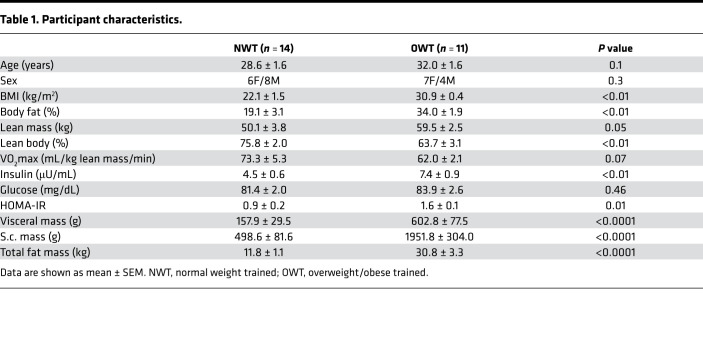
Participant characteristics.

## Version 1 02/22/2022

In-Press Preview

## Version 2 04/08/2022

Electronic publication
