# Supplementary material for: Acute aerobic exercise reveals that FAHFAs distinguish the metabolomes of overweight and normal-weight runners
Source: JCI Insight. 2022 Apr 8;7(7):e158037. doi: 10.1172/jci.insight.158037 (PMC9057596; doi:10.1172/jci.insight.158037)
Supplement: Supplemental data [file jciinsight-7-158037-s130.pdf]

A

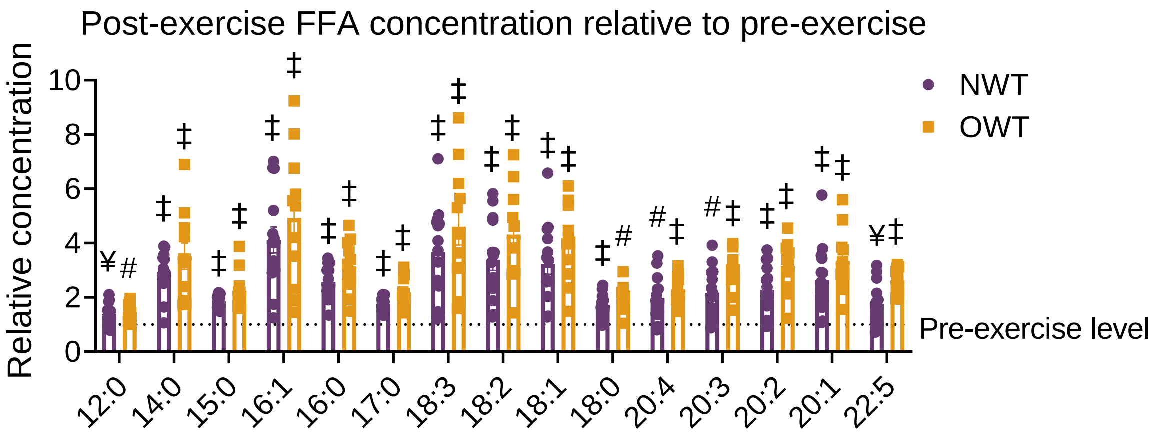

B

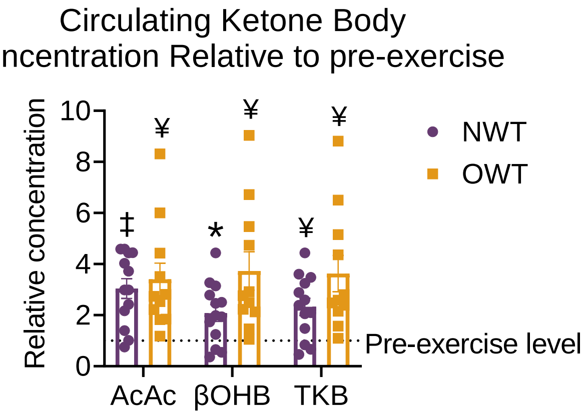

Supplemental Figure 1: Quantitation of lipids and total ketone bodies in NWT and OWT by targeted shotgun lipidomics. Concentration of (A) free fatty acid (FFA) species and (B) ketone bodies relative to pre-exercise. AcAc: acetoacetate; βOHB: beta-hydroxybutyrate; TKB: Total Ketone Bodies. *: p≤0.05; ¥: p≤0.01; #: p≤0.001; ‡: p≤0.0001. Significance symbols denote pre- to post-exercise comparison by Student's *t* test with Benjamini-Hochberg correction for multiple testing. Data represent mean ± SEM.
