# Supplementary material for: Acute aerobic exercise reveals that FAHFAs distinguish the metabolomes of overweight and normal-weight runners
Source: JCI Insight. 2022 Apr 8;7(7):e158037. doi: 10.1172/jci.insight.158037 (PMC9057596; doi:10.1172/jci.insight.158037)
Supplement: ICMJE disclosure forms [file jciinsight-7-158037-s131.pdf]

ICMJE DISCLOSURE FORM

**Date:**	2/10/2022
**Your Name:**	Alisa Nelson
**Manuscript Title:**	Acute aerobic exercise reveals FAHFAs distinguish the metabolomes of overweight and normal weight runners
**Manuscript Number (if known):**	158037-INS-CMED-TR-2

In the interest of transparency, we ask you to disclose all relationships/activities/interests listed below that are related to the content of your manuscript. "Related" means any relation with for-profit or not-for-profit third parties whose interests may be affected by the content of the manuscript. Disclosure represents a commitment to transparency and does not necessarily indicate a bias. If you are in doubt about whether to list a relationship/activity/interest, it is preferable that you do so.

The author's relationships/activities/interests should be defined broadly. For example, if your manuscript pertains to the epidemiology of hypertension, you should declare all relationships with manufacturers of antihypertensive medication, even if that medication is not mentioned in the manuscript.

In item #1 below, report all support for the work reported in this manuscript without time limit. For all other items, the time frame for disclosure is the past 36 months.

		**Name all entities with whom you have this relationship or indicate none (add rows as needed)**	**Specifications/Comments (e.g., if payments were made to you or to your institution)**
**Time frame: Since the initial planning of the work**			
1	All support for the present manuscript (e.g., funding, provision of study materials, medical writing, article processing charges, etc.) **No time limit for this item.**	☐ None	
		NIH DK091538	NIH TR000114
		NIH AG069781	NIH UL1TR002494
		NIH DK098203	Click the tab key to add additional rows.
**Time frame: past 36 months**			
**2**	Grants or contracts from any entity (if not indicated in item #1 above).	☒ **None**	
**3**	Royalties or licenses	☒ **None**	

		Name all entities with whom you have this relationship or indicate none (add rows as needed)	Specifications/Comments (e.g., if payments were made to you or to your institution)
**4**	Consulting fees	☒ **None**	
**5**	Payment or honoraria for lectures, presentations, speakers bureaus, manuscript writing or educational events	☒ **None**	
**6**	Payment for expert testimony	☒ **None**	
**7**	Support for attending meetings and/or travel	☒ **None**	
**8**	Patents planned, issued or pending	☒ **None**	
**9**	Participation on a Data Safety Monitoring Board or Advisory Board	☒ **None**	
**10**	Leadership or fiduciary role in other board, society, committee or advocacy group, paid or unpaid	☒ **None**	

		Name all entities with whom you have this relationship or indicate none (add rows as needed)	Specifications/Comments (e.g., if payments were made to you or to your institution)
**11**	Stock or stock options	☒ **None**	
**12**	Receipt of equipment, materials, drugs, medical writing, gifts or other services	☒ **None**	
**13**	Other financial or non-financial interests	☒ **None**	

Please place an "X" next to the following statement to indicate your agreement:

☒ I certify that I have answered every question and have not altered the wording of any of the questions on this form.

ICMJE DISCLOSURE FORM

Date: 2/10/2022

Your Name: Lisa Chow

Manuscript Title: Acute aerobic exercise reveals FAHFAs distinguish the metabolomes of overweight and normal weight runners

Manuscript Number (if known): 158037-INS-CMED-TR-2

In the interest of transparency, we ask you to disclose all relationships/activities/interests listed below that are related to the content of your manuscript. "Related" means any relation with for-profit or not-for-profit third parties whose interests may be affected by the content of the manuscript. Disclosure represents a commitment to transparency and does not necessarily indicate a bias. If you are in doubt about whether to list a relationship/activity/interest, it is preferable that you do so.

The author's relationships/activities/interests should be defined broadly. For example, if your manuscript pertains to the epidemiology of hypertension, you should declare all relationships with manufacturers of antihypertensive medication, even if that medication is not mentioned in the manuscript.

In item #1 below, report all support for the work reported in this manuscript without time limit. For all other items, the time frame for disclosure is the past 36 months.

		**Name all entities with whom you have this relationship or indicate none (add rows as needed)**	**Specifications/Comments (e.g., if payments were made to you or to your institution)**
**Time frame: Since the initial planning of the work**			
1	All support for the present manuscript (e.g., funding, provision of study materials, medical writing, article processing charges, etc.) **No time limit for this item.**	☐ None	
		NIH DK091538	NIH TR000114
		NIH AG069781	NIH UL1TR002494
		NIH DK098203	Click the tab key to add additional rows.
**Time frame: past 36 months**			
**2**	Grants or contracts from any entity (if not indicated in item #1 above).	☒ **None**	
**3**	Royalties or licenses	☒ **None**	

		Name all entities with whom you have this relationship or indicate none (add rows as needed)	Specifications/Comments (e.g., if payments were made to you or to your institution)
**4**	Consulting fees	☒ **None**	
**5**	Payment or honoraria for lectures, presentations, speakers bureaus, manuscript writing or educational events	☒ **None**	
**6**	Payment for expert testimony	☒ **None**	
**7**	Support for attending meetings and/or travel	☒ **None**	
**8**	Patents planned, issued or pending	☒ **None**	
**9**	Participation on a Data Safety Monitoring Board or Advisory Board	☒ **None**	
**10**	Leadership or fiduciary role in other board, society, committee or advocacy group, paid or unpaid	☒ **None**	

		Name all entities with whom you have this relationship or indicate none (add rows as needed)	Specifications/Comments (e.g., if payments were made to you or to your institution)
**11**	Stock or stock options	☒ **None**	
**12**	Receipt of equipment, materials, drugs, medical writing, gifts or other services	☒ **None**	
**13**	Other financial or non-financial interests	☒ **None**	

Please place an "X" next to the following statement to indicate your agreement:

☒ I certify that I have answered every question and have not altered the wording of any of the questions on this form.

ICMJE DISCLOSURE FORM

**Date:**	2/10/2022
**Your Name:**	Jacob Gillingham
**Manuscript Title:**	Acute aerobic exercise reveals FAHFAs distinguish the metabolomes of overweight and normal weight runners
**Manuscript Number (if known):**	158037-INS-CMED-TR-2

In the interest of transparency, we ask you to disclose all relationships/activities/interests listed below that are related to the content of your manuscript. "Related" means any relation with for-profit or not-for-profit third parties whose interests may be affected by the content of the manuscript. Disclosure represents a commitment to transparency and does not necessarily indicate a bias. If you are in doubt about whether to list a relationship/activity/interest, it is preferable that you do so.

The author's relationships/activities/interests should be defined broadly. For example, if your manuscript pertains to the epidemiology of hypertension, you should declare all relationships with manufacturers of antihypertensive medication, even if that medication is not mentioned in the manuscript.

In item #1 below, report all support for the work reported in this manuscript without time limit. For all other items, the time frame for disclosure is the past 36 months.

		**Name all entities with whom you have this relationship or indicate none (add rows as needed)**	**Specifications/Comments (e.g., if payments were made to you or to your institution)**
**Time frame: Since the initial planning of the work**			
1	All support for the present manuscript (e.g., funding, provision of study materials, medical writing, article processing charges, etc.) **No time limit for this item.**	☐ None	
		NIH DK091538	NIH TR000114
		NIH AG069781	NIH UL1TR002494
		NIH DK098203	Click the tab key to add additional rows.
**Time frame: past 36 months**			
**2**	Grants or contracts from any entity (if not indicated in item #1 above).	☒ **None**	
**3**	Royalties or licenses	☒ **None**	

		Name all entities with whom you have this relationship or indicate none (add rows as needed)	Specifications/Comments (e.g., if payments were made to you or to your institution)
**4**	Consulting fees	☒ **None**	
**5**	Payment or honoraria for lectures, presentations, speakers bureaus, manuscript writing or educational events	☒ **None**	
**6**	Payment for expert testimony	☒ **None**	
**7**	Support for attending meetings and/or travel	☒ **None**	
**8**	Patents planned, issued or pending	☒ **None**	
**9**	Participation on a Data Safety Monitoring Board or Advisory Board	☒ **None**	
**10**	Leadership or fiduciary role in other board, society, committee or advocacy group, paid or unpaid	☒ **None**	

		Name all entities with whom you have this relationship or indicate none (add rows as needed)	Specifications/Comments (e.g., if payments were made to you or to your institution)
**11**	Stock or stock options	☒ **None**	
**12**	Receipt of equipment, materials, drugs, medical writing, gifts or other services	☒ **None**	
**13**	Other financial or non-financial interests	☒ **None**	

Please place an "X" next to the following statement to indicate your agreement:

☒ I certify that I have answered every question and have not altered the wording of any of the questions on this form.

ICMJE DISCLOSURE FORM

**Date:**	2/10/2022
**Your Name:**	Meixia Pan
**Manuscript Title:**	Acute aerobic exercise reveals FAHFAs distinguish the metabolomes of overweight and normal weight runners
**Manuscript Number (if known):**	158037-INS-CMED-TR-2

In the interest of transparency, we ask you to disclose all relationships/activities/interests listed below that are related to the content of your manuscript. "Related" means any relation with for-profit or not-for-profit third parties whose interests may be affected by the content of the manuscript. Disclosure represents a commitment to transparency and does not necessarily indicate a bias. If you are in doubt about whether to list a relationship/activity/interest, it is preferable that you do so.

The author's relationships/activities/interests should be defined broadly. For example, if your manuscript pertains to the epidemiology of hypertension, you should declare all relationships with manufacturers of antihypertensive medication, even if that medication is not mentioned in the manuscript.

In item #1 below, report all support for the work reported in this manuscript without time limit. For all other items, the time frame for disclosure is the past 36 months.

		**Name all entities with whom you have this relationship or indicate none (add rows as needed)**	**Specifications/Comments (e.g., if payments were made to you or to your institution)**
**Time frame: Since the initial planning of the work**			
1	All support for the present manuscript (e.g., funding, provision of study materials, medical writing, article processing charges, etc.) **No time limit for this item.**	☐ None	
		NIH DK091538	NIH TR000114
		NIH AG069781	NIH UL1TR002494
		NIH DK098203	Click the tab key to add additional rows.
**Time frame: past 36 months**			
**2**	Grants or contracts from any entity (if not indicated in item #1 above).	☒ **None**	
**3**	Royalties or licenses	☒ **None**	

		Name all entities with whom you have this relationship or indicate none (add rows as needed)	Specifications/Comments (e.g., if payments were made to you or to your institution)
**4**	Consulting fees	☒ **None**	
**5**	Payment or honoraria for lectures, presentations, speakers bureaus, manuscript writing or educational events	☒ **None**	
**6**	Payment for expert testimony	☒ **None**	
**7**	Support for attending meetings and/or travel	☒ **None**	
**8**	Patents planned, issued or pending	☒ **None**	
**9**	Participation on a Data Safety Monitoring Board or Advisory Board	☒ **None**	
**10**	Leadership or fiduciary role in other board, society, committee or advocacy group, paid or unpaid	☒ **None**	

		Name all entities with whom you have this relationship or indicate none (add rows as needed)	Specifications/Comments (e.g., if payments were made to you or to your institution)
**11**	Stock or stock options	☒ **None**	
**12**	Receipt of equipment, materials, drugs, medical writing, gifts or other services	☒ **None**	
**13**	Other financial or non-financial interests	☒ **None**	

Please place an "X" next to the following statement to indicate your agreement:

☒ I certify that I have answered every question and have not altered the wording of any of the questions on this form.

ICMJE DISCLOSURE FORM

**Date:**	2/10/2022
**Your Name:**	Curtis C. Hughey
**Manuscript Title:**	Acute aerobic exercise reveals FAHFAs distinguish the metabolomes of overweight and normal weight runners
**Manuscript Number (if known):**	158037-INS-CMED-TR-2

In the interest of transparency, we ask you to disclose all relationships/activities/interests listed below that are related to the content of your manuscript. "Related" means any relation with for-profit or not-for-profit third parties whose interests may be affected by the content of the manuscript. Disclosure represents a commitment to transparency and does not necessarily indicate a bias. If you are in doubt about whether to list a relationship/activity/interest, it is preferable that you do so.

The author's relationships/activities/interests should be defined broadly. For example, if your manuscript pertains to the epidemiology of hypertension, you should declare all relationships with manufacturers of antihypertensive medication, even if that medication is not mentioned in the manuscript.

In item #1 below, report all support for the work reported in this manuscript without time limit. For all other items, the time frame for disclosure is the past 36 months.

		Name all entities with whom you have this relationship or indicate none (add rows as needed)	Specifications/Comments (e.g., if payments were made to you or to your institution)
**Time frame: Since the initial planning of the work**			
1	All support for the present manuscript (e.g., funding, provision of study materials, medical writing, article processing charges, etc.) **No time limit for this item.**	☐ None	
		NIH DK091538	NIH TR000114
		NIH AG069781	NIH UL1TR002494
		NIH DK098203	Click the tab key to add additional rows.
**Time frame: past 36 months**			
**2**	Grants or contracts from any entity (if not indicated in item #1 above).	☒ **None**	
**3**	Royalties or licenses	☒ **None**	

		Name all entities with whom you have this relationship or indicate none (add rows as needed)	Specifications/Comments (e.g., if payments were made to you or to your institution)
4	Consulting fees	☒ **None**	
5	Payment or honoraria for lectures, presentations, speakers bureaus, manuscript writing or educational events	☒ **None**	
6	Payment for expert testimony	☒ **None**	
7	Support for attending meetings and/or travel	☒ **None**	
8	Patents planned, issued or pending	☒ **None**	
9	Participation on a Data Safety Monitoring Board or Advisory Board	☒ **None**	
10	Leadership or fiduciary role in other board, society, committee or advocacy group, paid or unpaid	☒ **None**	

		Name all entities with whom you have this relationship or indicate none (add rows as needed)	Specifications/Comments (e.g., if payments were made to you or to your institution)
**11**	Stock or stock options	☒ **None**	
**12**	Receipt of equipment, materials, drugs, medical writing, gifts or other services	☒ **None**	
**13**	Other financial or non-financial interests	☒ **None**	

Please place an "X" next to the following statement to indicate your agreement:

☒ I certify that I have answered every question and have not altered the wording of any of the questions on this form.

ICMJE DISCLOSURE FORM

**Date:**	2/10/2022
**Your Name:**	Michael Evans
**Manuscript Title:**	Acute aerobic exercise reveals FAHFAs distinguish the metabolomes of overweight and normal weight runners
**Manuscript Number (if known):**	158037-INS-CMED-TR-2

In the interest of transparency, we ask you to disclose all relationships/activities/interests listed below that are related to the content of your manuscript. "Related" means any relation with for-profit or not-for-profit third parties whose interests may be affected by the content of the manuscript. Disclosure represents a commitment to transparency and does not necessarily indicate a bias. If you are in doubt about whether to list a relationship/activity/interest, it is preferable that you do so.

The author's relationships/activities/interests should be defined broadly. For example, if your manuscript pertains to the epidemiology of hypertension, you should declare all relationships with manufacturers of antihypertensive medication, even if that medication is not mentioned in the manuscript.

In item #1 below, report all support for the work reported in this manuscript without time limit. For all other items, the time frame for disclosure is the past 36 months.

		**Name all entities with whom you have this relationship or indicate none (add rows as needed)**	**Specifications/Comments (e.g., if payments were made to you or to your institution)**
**Time frame: Since the initial planning of the work**			
1	All support for the present manuscript (e.g., funding, provision of study materials, medical writing, article processing charges, etc.) **No time limit for this item.**	☐ None	
		NIH UL1TR002494	Institution
			Click the tab key to add additional rows.
**Time frame: past 36 months**			
**2**	Grants or contracts from any entity (if not indicated in item #1 above).	☒ **None**	
**3**	Royalties or licenses	☒ **None**	

		Name all entities with whom you have this relationship or indicate none (add rows as needed)	Specifications/Comments (e.g., if payments were made to you or to your institution)
**4**	Consulting fees	☒ **None**	
**5**	Payment or honoraria for lectures, presentations, speakers bureaus, manuscript writing or educational events	☒ **None**	
**6**	Payment for expert testimony	☒ **None**	
**7**	Support for attending meetings and/or travel	☒ **None**	
**8**	Patents planned, issued or pending	☒ **None**	
**9**	Participation on a Data Safety Monitoring Board or Advisory Board	☒ **None**	
**10**	Leadership or fiduciary role in other board, society, committee or advocacy group, paid or unpaid	☒ **None**	

		Name all entities with whom you have this relationship or indicate none (add rows as needed)	Specifications/Comments (e.g., if payments were made to you or to your institution)
**11**	Stock or stock options	☒ **None**	
**12**	Receipt of equipment, materials, drugs, medical writing, gifts or other services	☒ **None**	
**13**	Other financial or non-financial interests	☒ **None**	

Please place an "X" next to the following statement to indicate your agreement:

☒ I certify that I have answered every question and have not altered the wording of any of the questions on this form.

ICMJE DISCLOSURE FORM

**Date:**	2/10/2022
**Your Name:**	Xianlin Han
**Manuscript Title:**	Acute aerobic exercise reveals FAHFAs distinguish the metabolomes of overweight and normal weight runners
**Manuscript Number (if known):**	158037-INS-CMED-TR-2

In the interest of transparency, we ask you to disclose all relationships/activities/interests listed below that are related to the content of your manuscript. "Related" means any relation with for-profit or not-for-profit third parties whose interests may be affected by the content of the manuscript. Disclosure represents a commitment to transparency and does not necessarily indicate a bias. If you are in doubt about whether to list a relationship/activity/interest, it is preferable that you do so.

The author's relationships/activities/interests should be defined broadly. For example, if your manuscript pertains to the epidemiology of hypertension, you should declare all relationships with manufacturers of antihypertensive medication, even if that medication is not mentioned in the manuscript.

In item #1 below, report all support for the work reported in this manuscript without time limit. For all other items, the time frame for disclosure is the past 36 months.

		**Name all entities with whom you have this relationship or indicate none (add rows as needed)**	**Specifications/Comments (e.g., if payments were made to you or to your institution)**
**Time frame: Since the initial planning of the work**			
1	All support for the present manuscript (e.g., funding, provision of study materials, medical writing, article processing charges, etc.) **No time limit for this item.**	☐ None	
		NIH DK091538	NIH TR000114
		NIH AG069781	NIH UL1TR002494
		NIH DK098203	NIH RF1AG061729
**Time frame: past 36 months**			
**2**	Grants or contracts from any entity (if not indicated in item #1 above).	☒ **None**	
**3**	Royalties or licenses	☒ **None**	

		Name all entities with whom you have this relationship or indicate none (add rows as needed)	Specifications/Comments (e.g., if payments were made to you or to your institution)
**4**	Consulting fees	☒ **None**	
**5**	Payment or honoraria for lectures, presentations, speakers bureaus, manuscript writing or educational events	☒ **None**	
**6**	Payment for expert testimony	☒ **None**	
**7**	Support for attending meetings and/or travel	☒ **None**	
**8**	Patents planned, issued or pending	☒ **None**	
**9**	Participation on a Data Safety Monitoring Board or Advisory Board	☒ **None**	
**10**	Leadership or fiduciary role in other board, society, committee or advocacy group, paid or unpaid	☒ **None**	

		Name all entities with whom you have this relationship or indicate none (add rows as needed)	Specifications/Comments (e.g., if payments were made to you or to your institution)
**11**	Stock or stock options	☒ **None**	
**12**	Receipt of equipment, materials, drugs, medical writing, gifts or other services	☒ **None**	
**13**	Other financial or non-financial interests	☒ **None**	

Please place an "X" next to the following statement to indicate your agreement:

☒ I certify that I have answered every question and have not altered the wording of any of the questions on this form.

ICMJE DISCLOSURE FORM

**Date:**	2/11/2022
**Your Name:**	Patrycja Puchalsa
**Manuscript Title:**	Acute aerobic exercise reveals FAHFAs distinguish the metabolomes of overweight and normal weight runners
**Manuscript Number (if known):**	158037-INS-CMED-TR-2

In the interest of transparency, we ask you to disclose all relationships/activities/interests listed below that are related to the content of your manuscript. "Related" means any relation with for-profit or not-for-profit third parties whose interests may be affected by the content of the manuscript. Disclosure represents a commitment to transparency and does not necessarily indicate a bias. If you are in doubt about whether to list a relationship/activity/interest, it is preferable that you do so.

The author's relationships/activities/interests should be defined broadly. For example, if your manuscript pertains to the epidemiology of hypertension, you should declare all relationships with manufacturers of antihypertensive medication, even if that medication is not mentioned in the manuscript.

In item #1 below, report all support for the work reported in this manuscript without time limit. For all other items, the time frame for disclosure is the past 36 months.

		Name all entities with whom you have this relationship or indicate none (add rows as needed)	Specifications/Comments (e.g., if payments were made to you or to your institution)
**Time frame: Since the initial planning of the work**			
1	All support for the present manuscript (e.g., funding, provision of study materials, medical writing, article processing charges, etc.) **No time limit for this item.**	☐ None	
		NIH DK091538	NIH TR000114
		NIH AG069781	NIH UL1TR002494
		NIH DK098203	Click the tab key to add additional rows.
**Time frame: past 36 months**			
**2**	Grants or contracts from any entity (if not indicated in item #1 above).	☒ **None**	
**3**	Royalties or licenses	☒ **None**	

		Name all entities with whom you have this relationship or indicate none (add rows as needed)	Specifications/Comments (e.g., if payments were made to you or to your institution)
**4**	Consulting fees	☒ **None**	
**5**	Payment or honoraria for lectures, presentations, speakers bureaus, manuscript writing or educational events	☒ **None**	
**6**	Payment for expert testimony	☒ **None**	
**7**	Support for attending meetings and/or travel	☒ **None**	
**8**	Patents planned, issued or pending	☒ **None**	
**9**	Participation on a Data Safety Monitoring Board or Advisory Board	☒ **None**	
**10**	Leadership or fiduciary role in other board, society, committee or advocacy group, paid or unpaid	☒ **None**	

		Name all entities with whom you have this relationship or indicate none (add rows as needed)	Specifications/Comments (e.g., if payments were made to you or to your institution)
**11**	Stock or stock options	☒ **None**	
**12**	Receipt of equipment, materials, drugs, medical writing, gifts or other services	☒ **None**	
**13**	Other financial or non-financial interests	☒ **None**	

Please place an "X" next to the following statement to indicate your agreement:

☒ I certify that I have answered every question and have not altered the wording of any of the questions on this form.

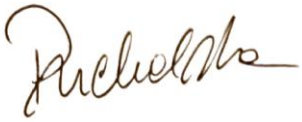

ICMJE DISCLOSURE FORM

**Date:**	2/10/2022
**Your Name:**	Peter Crawford
**Manuscript Title:**	Acute aerobic exercise reveals FAHFAs distinguish the metabolomes of overweight and normal weight runners
**Manuscript Number (if known):**	158037-INS-CMED-TR-2

In the interest of transparency, we ask you to disclose all relationships/activities/interests listed below that are related to the content of your manuscript. "Related" means any relation with for-profit or not-for-profit third parties whose interests may be affected by the content of the manuscript. Disclosure represents a commitment to transparency and does not necessarily indicate a bias. If you are in doubt about whether to list a relationship/activity/interest, it is preferable that you do so.

The author's relationships/activities/interests should be defined broadly. For example, if your manuscript pertains to the epidemiology of hypertension, you should declare all relationships with manufacturers of antihypertensive medication, even if that medication is not mentioned in the manuscript.

In item #1 below, report all support for the work reported in this manuscript without time limit. For all other items, the time frame for disclosure is the past 36 months.

		**Name all entities with whom you have this relationship or indicate none (add rows as needed)**	**Specifications/Comments (e.g., if payments were made to you or to your institution)**
**Time frame: Since the initial planning of the work**			
1	All support for the present manuscript (e.g., funding, provision of study materials, medical writing, article processing charges, etc.) **No time limit for this item.**	☐ None	
		NIH DK091538	NIH TR000114
		NIH AG069781	NIH UL1TR002494
		NIH DK098203	Click the tab key to add additional rows.
**Time frame: past 36 months**			
**2**	Grants or contracts from any entity (if not indicated in item #1 above).	☐ **None**	
		UMN - Academic Investment Research Program	
**3**	Royalties or licenses	☒ **None**	

		Name all entities with whom you have this relationship or indicate none (add rows as needed)	Specifications/Comments (e.g., if payments were made to you or to your institution)
**4**	Consulting fees	☐ **None**	
		Pfizer Inc.	Payment to me
		Janssen Cardiovascular and Metabolic Health	Payment to me
		Abbott Diabetes Care	Payment to me
**5**	Payment or honoraria for lectures, presentations, speakers bureaus, manuscript writing or educational events	☐ **None**	
		All academic (lectures): 15 universities and academic societies	Payments from a small subset to me
**6**	Payment for expert testimony	☒ **None**	
**7**	Support for attending meetings and/or travel	☒ **None**	
**8**	Patents planned, issued or pending	☒ **None**	
**9**	Participation on a Data Safety Monitoring Board or Advisory Board	☒ **None**	
**10**	Leadership or fiduciary role in other board, society, committee or advocacy group, paid or unpaid	☒ **None**	

		Name all entities with whom you have this relationship or indicate none (add rows as needed)	Specifications/Comments (e.g., if payments were made to you or to your institution)
**11**	Stock or stock options	☒ **None**	
**12**	Receipt of equipment, materials, drugs, medical writing, gifts or other services	☒ **None**	
**13**	Other financial or non-financial interests	☒ **None**	

Please place an "X" next to the following statement to indicate your agreement:

☒ I certify that I have answered every question and have not altered the wording of any of the questions on this form.

ICMJE DISCLOSURE FORM

**Date:**	2/10/2022
**Your Name:**	Chad Myers
**Manuscript Title:**	Acute aerobic exercise reveals FAHFAs distinguish the metabolomes of overweight and normal weight runners
**Manuscript Number (if known):**	158037-INS-CMED-TR-2

In the interest of transparency, we ask you to disclose all relationships/activities/interests listed below that are related to the content of your manuscript. "Related" means any relation with for-profit or not-for-profit third parties whose interests may be affected by the content of the manuscript. Disclosure represents a commitment to transparency and does not necessarily indicate a bias. If you are in doubt about whether to list a relationship/activity/interest, it is preferable that you do so.

The author's relationships/activities/interests should be defined broadly. For example, if your manuscript pertains to the epidemiology of hypertension, you should declare all relationships with manufacturers of antihypertensive medication, even if that medication is not mentioned in the manuscript.

In item #1 below, report all support for the work reported in this manuscript without time limit. For all other items, the time frame for disclosure is the past 36 months.

		Name all entities with whom you have this relationship or indicate none (add rows as needed)	Specifications/Comments (e.g., if payments were made to you or to your institution)
**Time frame: Since the initial planning of the work**			
1	All support for the present manuscript (e.g., funding, provision of study materials, medical writing, article processing charges, etc.) **No time limit for this item.**	☐ None	
		NIH DK091538	NIH TR000114
		NIH AG069781	NIH UL1TR002494
		NIH DK098203	Click the tab key to add additional rows.
**Time frame: past 36 months**			
**2**	Grants or contracts from any entity (if not indicated in item #1 above).	☒ **None**	
**3**	Royalties or licenses	☒ **None**	

		Name all entities with whom you have this relationship or indicate none (add rows as needed)	Specifications/Comments (e.g., if payments were made to you or to your institution)
**4**	Consulting fees	☒ **None**	
**5**	Payment or honoraria for lectures, presentations, speakers bureaus, manuscript writing or educational events	☒ **None**	
**6**	Payment for expert testimony	☒ **None**	
**7**	Support for attending meetings and/or travel	☒ **None**	
**8**	Patents planned, issued or pending	☒ **None**	
**9**	Participation on a Data Safety Monitoring Board or Advisory Board	☒ **None**	
**10**	Leadership or fiduciary role in other board, society, committee or advocacy group, paid or unpaid	☒ **None**	

		Name all entities with whom you have this relationship or indicate none (add rows as needed)	Specifications/Comments (e.g., if payments were made to you or to your institution)
**11**	Stock or stock options	☒ **None**	
**12**	Receipt of equipment, materials, drugs, medical writing, gifts or other services	☒ **None**	
**13**	Other financial or non-financial interests	☒ **None**	

Please place an "X" next to the following statement to indicate your agreement:

☐ I certify that I have answered every question and have not altered the wording of any of the questions on this form.

ICMJE DISCLOSURE FORM

**Date:**	2/10/2022
**Your Name:**	David B. Stagg
**Manuscript Title:**	Acute aerobic exercise reveals FAHFAs distinguish the metabolomes of overweight and normal weight runners
**Manuscript Number (if known):**	158037-INS-CMED-TR-2

In the interest of transparency, we ask you to disclose all relationships/activities/interests listed below that are related to the content of your manuscript. "Related" means any relation with for-profit or not-for-profit third parties whose interests may be affected by the content of the manuscript. Disclosure represents a commitment to transparency and does not necessarily indicate a bias. If you are in doubt about whether to list a relationship/activity/interest, it is preferable that you do so.

The author's relationships/activities/interests should be defined broadly. For example, if your manuscript pertains to the epidemiology of hypertension, you should declare all relationships with manufacturers of antihypertensive medication, even if that medication is not mentioned in the manuscript.

In item #1 below, report all support for the work reported in this manuscript without time limit. For all other items, the time frame for disclosure is the past 36 months.

		Name all entities with whom you have this relationship or indicate none (add rows as needed)	Specifications/Comments (e.g., if payments were made to you or to your institution)
**Time frame: Since the initial planning of the work**			
1	All support for the present manuscript (e.g., funding, provision of study materials, medical writing, article processing charges, etc.) **No time limit for this item.**	☐ None	
		NIH DK091538	NIH TR000114
		NIH AG069781	NIH UL1TR002494
		NIH DK098203	Click the tab key to add additional rows.
**Time frame: past 36 months**			
**2**	Grants or contracts from any entity (if not indicated in item #1 above).	☒ **None**	
**3**	Royalties or licenses	☒ **None**	

		Name all entities with whom you have this relationship or indicate none (add rows as needed)	Specifications/Comments (e.g., if payments were made to you or to your institution)
**4**	Consulting fees	☒ **None**	
**5**	Payment or honoraria for lectures, presentations, speakers bureaus, manuscript writing or educational events	☒ **None**	
**6**	Payment for expert testimony	☒ **None**	
**7**	Support for attending meetings and/or travel	☒ **None**	
**8**	Patents planned, issued or pending	☒ **None**	
**9**	Participation on a Data Safety Monitoring Board or Advisory Board	☒ **None**	
**10**	Leadership or fiduciary role in other board, society, committee or advocacy group, paid or unpaid	☒ **None**	

		Name all entities with whom you have this relationship or indicate none (add rows as needed)	Specifications/Comments (e.g., if payments were made to you or to your institution)
**11**	Stock or stock options	☒ **None**	
**12**	Receipt of equipment, materials, drugs, medical writing, gifts or other services	☒ **None**	
**13**	Other financial or non-financial interests	☒ **None**	

Please place an "X" next to the following statement to indicate your agreement:

☒ I certify that I have answered every question and have not altered the wording of any of the questions on this form.

STROBE Statement—checklist of items that should be included in reports of observational studies

	Item No.	Recommendation	Page No.	Relevant text from manuscript
**Title and abstract**	1	(*a*) Indicate the study's design with a commonly used term in the title or the abstract	3	"**Methods**. Serum samples were collected from overweight trained (OWT) and normal weight trained (NWT) runners prior to and immediately after a supervised 90-minute treadmill run at 60% VO2max (NWT = 14, OWT = 11) in a cross-sectional study."
		(*b*) Provide in the abstract an informative and balanced summary of what was done and what was found	3	"**Methods**. Serum samples were collected from overweight trained (OWT) and normal weight trained (NWT) runners prior to and immediately after a supervised 90-minute treadmill run…" **Conclusion**. These findings in overweight human participants and healthy controls indicate that exercise-provoked changes in FAHFAs distinguish normal weight from overweight individuals and could predict VO2max.
**Introduction**				
Background/rationale	2	Explain the scientific background and rationale for the investigation being reported	4	"The modulating effect of exercise on insulin sensitivity makes it an important context to

				study the differential impact of fitness versus BMI on the metabolome. However, the effect of exercise on metabolism has largely been constrained to examining normal weight trained (NWT) runners or overweight/obese subjects trained from a sedentary state, whereas the population of overweight/obese trained (OWT) runners remains understudied.”
Objectives	3	State specific objectives, including any prespecified hypotheses	5	“…here we make the novel comparison between NWT and OWT regarding metabolome differences in the resting state and in response to acute aerobic exercise”
**Methods**				
Study design	4	Present key elements of study design early in the paper	5	“Using serum samples from NWT and OWT participants pre- and post-acute aerobic exercise, we utilized hydrophilic interaction chromatography with negative electrospray ionization (HILIC-ESI(-)-MS/MS) and reverse phase chromatography with positive electrospray ionization (RP-ESI(+)-MS/MS) to profile

				serum metabolites (42). NWT and OWT populations were matched for age, gender, fitness level, and the absence of clinical insulin resistance.”
Setting	5	Describe the setting, locations, and relevant dates, including periods of recruitment, exposure, follow-up, and data collection	19-20	“Study participant details. We recruited OWT (n=11) and NWT (n=14) participants who self-reported aerobic exercise (3-5 sessions/week) from the Twin Cities metro area between July 2014 and April 2017.” “Blood was drawn after an 8 hour fast to measure insulin and glucose levels to calculate insulin sensitivity…” “The acute aerobic exercise intervention was scheduled at least 1 week after the VO2max testing to minimize influence of the strenuous exercise from the VO2 max test. Subjects were instructed to avoid intentional exercise for two days before the second visit and arrive after an overnight fast (at least 8 hours).”
Participants	6	(*a*) *Cohort study*—Give the eligibility criteria, and the sources and methods of selection of participants. Describe methods of follow-up<br>*Case-control study*—Give the eligibility criteria, and the sources and methods of case ascertainment and control selection. Give the rationale for the choice of cases and controls	19	“Study participant details. We recruited OWT (n=11) and NWT (n=14) participants who self-reported aerobic exercise (3-5 sessions/week) from the

		Cross-sectional study—Give the eligibility criteria, and the sources and methods of selection of participants		Twin Cities metro area between July 2014 and April 2017. We preferentially recruited participants from recent running events, to ensure that they are capable to complete a prolonged (90 minute) run. Inclusion criteria were: 1) Age 18-40 years, and 2) Regular aerobic exercise, preferably running, at least 3-5 sessions/week. Individuals with 1) Self-reported clinically significant medical issues (for example diabetes, cardiovascular disease, uncontrolled pulmonary disease), 2) abnormal EKG indicating cardiac disease (study EKG performed) and 3) current pregnancy (screening pregnancy test performed) were excluded. Participants were recruited with the goal to achieve similarity in age and sex between the two groups."
		(*b*) *Cohort study*—For matched studies, give matching criteria and number of exposed and unexposed<br>*Case-control study*—For matched studies, give matching criteria and the number of controls per case		
Variables	7	Clearly define all outcomes, exposures, predictors, potential confounders, and effect modifiers. Give diagnostic criteria, if applicable	7	"The study involved a single 90-minute run at 60% of individual VO2max, after overnight fast (at

				least 8 hours). During the run, participants wore a heart rate monitor; study staff directly supervised each participant to ensure they maintained a heart rate consistent with 60% VO2max exertion. Blood samples were collected immediately pre- and post-running bout.”
Data sources/ measurement	8*	For each variable of interest, give sources of data and details of methods of assessment (measurement). Describe comparability of assessment methods if there is more than one group	19-20	“Blood was drawn after an 8 hour fast to measure insulin and glucose levels to calculate insulin sensitivity...” “Body composition was measured by DXA.” “Maximum oxygen consumption (VO2 max) was evaluated by indirect calorimetry using one of two metabolic carts, either at the Human Performance Teaching Laboratory (Ultima Medgraphics CPX-D, Medical Graphics Corporation, St. Paul, MN) or Masonic Clinical Research Unit (ParvoMedics TrueOne 2400 – OUSW 4.3.4 (20160202), Sandy, UT, USA). Fitness was quantified by VO2max, normalized for lean mass.” “For the supervised exercise bout, all subjects ran

				for 90 minutes on a treadmill.” “Plasma samples were collected pre-and post the acute aerobic exercise intervention.”
Bias	9	Describe any efforts to address potential sources of bias	19	“Participants were recruited with the goal to achieve similarity in age and sex between the two groups.”
Study size	10	Explain how the study size was arrived at	19	“We recruited OWT (n=11) and NWT (n=14) participants who self-reported aerobic exercise (3-5 sessions/week) from the Twin Cities metro area between July 2014 and April 2017.”

Continued on next page

Quantitative variables	11	Explain how quantitative variables were handled in the analyses. If applicable, describe which groupings were chosen and why	8, 22	“Data analyses were performed after ensuring the I.S. areas under the curve in all analyzed samples were consistent across the batch (RSD<10%).” “Differential abundance analysis was performed to analyze the effect of 1) BMI or 2) Acute exercise. Peaks with log2 fold change > 1 and p value < 0.05 were selected and evaluated using Compound Discoverer 2.1 visualization tools for quality of spectra, peak picking, and area integration.”
Statistical methods	12	(*a*) Describe all statistical methods, including those used to control for confounding	24	“*Statistics*. Descriptive data are expressed as mean and standard error (SEM) for continuous measures and N (%) for categorical measures (Table 1). Comparisons between the NWT and OWT groups were performed using paired t-test. Statistical significance was defined as p ≤0.05 due to the sample size and exploratory nature of this study. All statistical analyses of metabolite profile used Benjamini-Hochberg to adjust for multiple testing across metabolites (81). Cytokine analyses were performed using SAS 9.3 (SAS Institute, Cary NC). Principal Component Analysis used log10-

				transformed raw intensities and R packages FactoMineR and Factoextra…”
		(*b*) Describe any methods used to examine subgroups and interactions	24	“The relationships between exercise-related change in metabolite levels (difference in log2 concentrations between post- and pre-exercise) and pre-exercise BMI, total fat mass, visceral fat mass, and subcutaneous fat mass were examined using linear models of metabolite changes, in univariate models and in models including sex and age covariates. Effects are reported as model coefficients with 95% confidence intervals after correction for multiple testing.<br>For predictive models for BMI and VO2max, ridge regression was used to build models using glmnet library in R.”
		(*c*) Explain how missing data were addressed	N/A	
		(*d*) *Cohort study*—If applicable, explain how loss to follow-up was addressed<br>*Case-control study*—If applicable, explain how matching of cases and controls was addressed<br>*Cross-sectional study*—If applicable, describe analytical methods taking account of sampling strategy	24	“For predictive models for BMI and VO2max, ridge regression was used to build models using glmnet library in R. LASSO was also tested but was outperformed by ridge in terms of model generalizability, and thus, results from ridge are reported here. Due to small sample size, five unique

				models were generated and are summarized. Samples were randomly sampled without replacement for 5 unique training and testing sets of combined NWT and OWT (80/20 split), and the sampling was constrained to maintain the participant sex distribution in each split to remove sex differences as a confounding factor. For each training/test set split, the lambda parameter was tuned based on the training set only using 10-fold cross-validation.”
		(*e*) Describe any sensitivity analyses	N/A	
**Results**				
Participants	13*	(a) Report numbers of individuals at each stage of study—eg numbers potentially eligible, examined for eligibility, confirmed eligible, included in the study, completing follow-up, and analysed	7	“Participant characteristics and study design. We enrolled normal weight and overweight or obese individuals with a self-reported history of running 3-5 sessions per week for a minimum of 30 minutes. Trained, rather than sedentary, individuals were studied to limit the effects of a deconditioned acute stress response. The cohort consisted of twenty-five runners (OWT: n = 11, NWT: n = 14; Table 1).”
		(b) Give reasons for non-participation at each stage	N/A	
		(c) Consider use of a flow diagram	N/A	

Descriptive data	14*	(a) Give characteristics of study participants (eg demographic, clinical, social) and information on exposures and potential confounders	7	"The cohort consisted of twenty-five runners (OWT: n = 11, NWT: n = 14; Table 1). The two groups differ by body mass index (BMI) [kg/m2: 30.9 ± 0.4 versus 22.1 ± 1.5, p < 0.01] and body fat [%: 34.0 ± 1.9 versus 19.1 ± 3.1, p < 0.01]. However, the differences in covariates, such as age, sex distribution, and VO2max [ml/kg lean body mass/min] were not significantly different between the groups as determined by t-test. Though HOMA-IR was higher in the OWT group than the NWT group [1.6 ± 0.1 vs 0.9 ± 0.2, p = 0.01], the absolute value of HOMA-IR in the OWT remains within the range for normal individuals."
		(b) Indicate number of participants with missing data for each variable of interest	N/A	
		(c) *Cohort study*—Summarise follow-up time (eg, average and total amount)		
Outcome data	15*	*Cohort study*—Report numbers of outcome events or summary measures over time		
		Case-control study—Report numbers in each exposure category, or summary measures of exposure		
		Cross-sectional study—Report numbers of outcome events or summary measures	N/A	
Main results	16	(*a*) Give unadjusted estimates and, if applicable, confounder-adjusted estimates and their precision (eg, 95% confidence interval). Make clear which confounders were adjusted for and why they were included	24-25	"The relationships between exercise-related change in metabolite levels (difference in log2 concentrations between post- and pre-exercise) and pre-exercise BMI, total fat mass, visceral fat mass, and

				subcutaneous fat mass were examined using linear models of metabolite changes, in univariate models and in models including sex and age covariates. Effects are reported as model coefficients with 95% confidence intervals after correction for multiple testing." "Samples were randomly sampled without replacement for 5 unique training and testing sets of combined NWT and OWT (80/20 split), and the sampling was constrained to maintain the participant sex distribution in each split to remove sex differences as a confounding factor." "Additional models were trained with the inclusion of sex and age; however, these did not significantly improve model performance."
		(*b*) Report category boundaries when continuous variables were categorized	7	"The two groups differ by body mass index (BMI) [kg/m2: 30.9 ± 0.4 versus 22.1 ± 1.5, p < 0.01]…"
		(*c*) If relevant, consider translating estimates of relative risk into absolute risk for a meaningful time period	N/A	

Continued on next page

Other analyses	17	Report other analyses done—eg analyses of subgroups and interactions, and sensitivity analyses	9	"In addition to serum metabolomics, twelve samples (NWT = 6, OWT = 6) were used to measure cytokines pre- and post-exercise."
**Discussion**				
Key results	18	Summarise key results with reference to study objectives	8	"We performed four different comparisons: 1) NWT group: pre- versus post-exercise; 2) OWT group: pre- versus post-exercise; 3) Pre-exercise: NWT versus OWT groups; 4) Post-exercise: NWT versus OWT groups. After correcting for multiple testing by false discovery rate, exercise impacted the abundance of 349 and 241 metabolites in NWT or OWT groups, respectively (Figure 2A-B; Supplemental Tables 1, 2). On the other hand, comparison of metabolome between NWT and OWT either before or after running bout showed that BMI influenced only 53 and 159 differentiating metabolites, respectively (Figure 2C-D; Supplemental Tables 3, 4)."
Limitations	19	Discuss limitations of the study, taking into account sources of potential bias or imprecision. Discuss both direction and magnitude of any potential bias	18	"This study has several important considerations. We report the metabolomics shift in serum of well-trained individuals with normal and high BMI. Previous

				studies have demonstrated BMI incompletely characterizes metabolic health (39). Some individuals within the OWT group had very low body fat and their exercise-induced changes were minimal for the identified metabolic profile (Figure 2B). This cross-sectional study sought individuals with an established exercise habit, and did not acquire further details on training history, diet, or body composition prior to training. These factors need to be considered in future human studies. Due to the small sample size, additional studies of FAHFAs in both untrained and trained individuals are required to demonstrate reproducibility of the relationships among FAHFAs, cardiovascular fitness and long-term health outcomes.”
Interpretation	20	Give a cautious overall interpretation of results considering objectives, limitations, multiplicity of analyses, results from similar studies, and other relevant evidence	18	“This study showed FAHFAs and purine nucleosides significantly contributed to variation in VO2max after normalizing for lean body mass. Intriguingly, FAHFAs were negatively associated with visceral fat mass while inosine was positively associated with subcutaneous fat. These

				relationships may indicate competing metabolic impacts from specific adipose depots that influence overall metabolic health. Future studies to uncover the role of FAHFAs in both acute and chronic exercise may provide insight into adipose tissue remodeling in exercise and offer a node for therapeutic intervention.”
Generalisability	21	Discuss the generalisability (external validity) of the study results	18	“Exercise is a ‘formidable regulator of insulin sensitivity and overall systemic metabolism’ (14). Acute and chronic effects of exercise force adaptation in several systems including adipose tissue, skeletal muscle, and the liver. For this reason, exercise continues to be the most effective intervention for metabolic diseases, such as type 2 diabetes and cardiovascular disease, and could be an important strategy in preventing MHO to MUO conversion. This study showed FAHFAs and purine nucleosides significantly contributed to variation in VO2max after normalizing for lean body mass. Intriguingly, FAHFAs were negatively associated with visceral fat mass while inosine was positively associated with

				subcutaneous fat. These relationships may indicate competing metabolic impacts from specific adipose depots that influence overall metabolic health. Future studies to uncover the role of FAHFAs in both acute and chronic exercise may provide insight into adipose tissue remodeling in exercise and offer a node for therapeutic intervention.”
**Other information**				
Funding	22	Give the source of funding and the role of the funders for the present study and, if applicable, for the original study on which the present article is based	3	“**Funding**. NIH DK091538, AG069781, DK098203, TR000114, UL1TR002494”

*Give information separately for cases and controls in case-control studies and, if applicable, for exposed and unexposed groups in cohort and cross-sectional studies.

Note: An Explanation and Elaboration article discusses each checklist item and gives methodological background and published examples of transparent reporting. The STROBE checklist is best used in conjunction with this article (freely available on the Web sites of PLoS Medicine at http://www.plosmedicine.org/, Annals of Internal Medicine at http://www.annals.org/, and Epidemiology at http://www.epidem.com/). Information on the STROBE Initiative is available at www.strobe-statement.org.
